# Supplementary material for: Discovery of l-threonine transaldolases for enhanced biosynthesis of beta-hydroxylated amino acids
Source: Commun Biol. 2023 Sep 11;6:929. doi: 10.1038/s42003-023-05293-0 (PMC10495429; doi:10.1038/s42003-023-05293-0)
Supplement: Supplementary file 2 — Supplementary Material [file 42003_2023_5293_MOESM2_ESM.pdf]

Supporting Information

Discovery of L-threonine transaldolases for enhanced biosynthesis of beta-hydroxylated amino acids

Michaela A. Jones[1], Neil D. Butler[1], Shelby R. Anderson[1], Sean A. Wirt[1], Ishika Govil[1], Xinyi Lyu[2], Yinzhi Fang[2], and Aditya M. Kunjapur[1]*

[1]*Department of Chemical and Biomolecular Engineering, University of Delaware, Newark, DE 19716, United States*

[2]*Department of Chemistry and Biochemistry, University of Delaware, Newark, DE 19716, United States*

*Correspondence: kunjapur@udel.edu

I. Supplementary Methods: ***3***

In vitro TTA-ADH-PTDH Assay **3**

Timed quench assay for HPLC analysis **3**

Amino acid substrate Assay **3**

Synthesis of 2-nitro-β-OH-phenylalanine **4**

Mass spectrometry confirmation of β-OH nsAAs using in vitro TTA-ADH coupled assay **4**

II. Supplementary Tables ***6***

Table S1: Absorbance of investigated aldehydes at 340 nm in phosphate buffer. **6**

Table S2. Predicted attributes of selected threonine transaldolases screened in this study. **7**

Table S3. Strains and Plasmids used in this study. **8**

Table S4. Oligonucleotides used in this study. **10**

Table S5. DNA G-Blocks/Twist gene fragments for cloning in this study. Start codons for each gene are underlined. **12**

III. Supplementary Figures ***16***

Figure S1 .... **16**

Figure S2 .... **17**

Figure S3 .... **18**

Figure S4 .... **19**

Figure S5 .... **20**

Figure S6 .... **21**

Figure S7 .... **22**

Figure S8 .... **23**

Figure S9 .... **24**

Figure S10......25
Figure S11......26
Figure S12......27
Figure S13......28
Figure S14......29
Figure S15......30
Figure S16......31
Figure S17......32
Figure S18......33
Figure S19......34
Figure S20......35
Figure S21......36
Figure S22......37
Figure S23......38
Figure S24......39
Figure S25......40
Figure S26......41
Figure S27......42
Figure S28......43
Figure S29......47
Figure S30......48
Figure S31......49
Figure S32......50
Figure S33......51
IV. Supplementary References......***52***

I. Supplementary Methods:

In vitro TTA-ADH-PTDH Assay

To improve product yields, we coupled the TTA with ScADH as well as an engineered phosphite dehydrogenase (PTDH). The assay was performed as described in the main text with 100 μL reaction volumes in a 96-well plate for the TTA-ADH coupled assay with 10 mM **3** and the addition of PTDH to a final concentration of 0.1 mg/mL and phosphite to a final concentration of 20 mM. The reaction was left shaking at 1000 RPM with an orbital radius of 1.25 mm at 30 ºC overnight. The reactions were quenched after 20 h with a final concentration of 1% TFA. Supernatant was collected following centrifugation and submitted to HPLC as reported in the main text.

Timed quench assay for HPLC analysis

Confirmation of the rates from the ADH assay were performed using the TTA-ADH coupled in vitro assay using a reaction mixture as reported in the main text in a 96-well plate shaking at 1000 RPM with an orbital radius of 1.25 mm at 30 ºC. We chose to quench with 1% TFA at approximately 0, 1, 2, 5, 10, 20, and 40 minutes to measure reaction kinetics. The samples were then collected and submitted to the HPLC for analysis as reported in the main text.

Amino acid substrate Assay

Reaction mixtures were prepared in a 96-well plate with 100 μL of 100mM phosphate buffer pH 7.5, 0.4 mM PLP, 15 mM $MgCl_2$, and 100 mM of the specified amino acid substrate with the addition of 1.5 mM **3**, and 0.25 μM purified TTA. The plate was incubated shaking at 1000 RPM with an orbital radius of 1.25 mm at 30 ºC overnight. The reactions were quenched after a 20 h with a final concentration of 1% TFA. Supernatant was collected following centrifugation and submitted to HPLC as reported in the main text.

Synthesis of 2-nitro-β-OH-phenylalanine

Synthesis of a racemic mixture of 2-nitro-β-OH-phenylalanine was performed according to a procedure described previously for phenylserines[1]. Triethylamine (3.07 mL, 22 mmol, 4.4 equiv) was added to a solution of glycine (375 mg, 5.0 mmol, 1.0 equiv) in water (4.0 mL). 2-Nitrobenzaldehyde (1.51 g, 10 mmol, 2.0 equiv) was added portion-wise to this solution, and the mixture was stirred overnight at ~25 °C. The color of the reaction mixture gradually changed from clear and colorless to yellow-brown. Toluene (10 mL) was added, and the triethylamine was evaporated under vacuum. The crude product was diluted with water (15 mL), and the mixture was acidified to pH 2 with HCl (6 M). The acidified solution was stirred at ~25 °C for 3 h and partitioned against ethyl acetate (2 × 15 mL) to remove the unreacted 2-nitrobenzaldehyde. The aqueous layer was separated and neutralized to pH 6.0 with a saturated $NaHCO_3$ solution to precipitate the product. The mixture was stirred for 1 h at 0 °C, and the 2-nitro-β-OH-phenylalanine was triturated with methanol followed by methanol/dichloromethane (50%, v/v), and dried under vacuum to yield 2-nitro-β-OH-phenylalanine (221 mg, 20%, off-white solid) as a mixture of diastereomers (*anti:syn* = 4:1). ^{1}H NMR (400 MHz, D_2O) *anti*-isomer: δ 8.26 (d, *J* = 8.1 Hz, 1H), 8.03-7.87 (m, 2H), 7.75-7.67 (m, 1H), 5.86 (d, *J* = 3.8 Hz, 1H), 4.30 (d, *J* = 3.8 Hz, 1H); *syn*-isomer: δ 8.26 (d, *J* = 8.1 Hz, 1H), 8.03-7.87 (m, 2H), 7.75-7.67 (m, 1H), 6.02 (d, *J* = 4.3 Hz, 1H), 4.24 (d, *J* = 4.4 Hz, 1H).

LC-MS method for standard: Waters ACQUITY Premier column, 3.0 min run, flow rate=0.5 mL/min, 95%-5% water/acetonitrile, retention time=0.24 min, ESI.

Mass spectrometry confirmation of β-OH nsAAs using in vitro TTA-ADH coupled assay

Mass spectrometry (MS) measurements for small molecule metabolites were submitted to a Waters AQUITY Arc UPLC H-Class with a diode array coupled to a Waters AQUITY QDa Mass Detector. Metabolite compounds were analyzed using a Waters Cortecs UPLC C18 column with an initial mobile phase of solvent A/B = 95/5 (solvent A, water, 0.1% formic acid; solvent B, acetonitrile, 0.1% formic acid) for 5 min with a gradient elution from (A/B) 95/5 to 10/90 for 5-7 min, an isocratic flow at 10/90 for 7-10

min, then gradient from 10/90 to 95/5 for 10-10.5 min and a final isocratic step for 10-12 min. Flow rate was maintained at 1 mL min^{-1}.

II. Supplementary Tables

Table S1: Absorbance of investigated aldehydes at 340 nm in phosphate buffer.

Aldehyde	Abs at 1mM (340 nm)	Final concentration in ADH assay (mM)
**1**	0.2452	1
**2**	0.3799	1
**3**	0.4418	1
**4**	0.3092	1
**5**	4	0.25
**6**	0.2291	1
**7**	0.2612	1
**8**	0.2796	1
**9**	0.2412	1
**10**	0.6106	1
**11**	0.2952	1
**12**	0.7088	1
**13**	0.2328	1
**14**	0.244	1
**15**	0.3858	1
**16**	0.4201	1
**Compound**	**Abs at 340 nm**	**Final concentration in ADH assay (mM)**
**PLP**	0.4273	0.4
**NADH**	0.9133	0.5
**L-Thr**	0.2233	100

Table S2. Predicted attributes of selected threonine transaldolases screened in this study.

Threonine transaldolase	Accession Number	Host Organism	Class	Host Genome Assembly for antiSMASH	antiSMASH BGC Type	antiSMASH Most similar known cluster (% similarity)
ObiH	ARJ35753.1	*Pseudomonas fluorescens*	Bacteria	KX931446.1*	Obafluorin	100%
PiTTA	WP_095149064.1	*Pseudomonas_sp._Irchel_s3a18*	Bacteria	NZ_FYDV01000019.1	Obafluorin	85%
BsTTA	WP_060149112.1	*Burkholderia stagnalis*	Bacteria	NZ_QTPN01000035.1	Obafluorin	71%
CsTTA	WP_018749561.1	*Chitiniphilus shinanonensis DSM 23277*	Bacteria	NZ_KB895358.1	Obafluorin	85%
BuTTA	WP_080410754.1	*Burkholderia ubonensis*	Bacteria	NZ_MECN01000006.1	N/A	
StTTA	WP_101279775.1	*Streptomyces (multi-species)*	Bacteria	NZ_CP031742.1	N/A	
TmTTA	WP_188596100	*Thermocladium modestius*	Archaea	NZ_BMNL01000002.1	N/A	
RaTTA	GIH11859	*Rugosimonospora africana*	Bacteria	BONZ01000001.1	Spicamycin	27%
SNTTA	ADZ45329	*Streptomyces sp. NRRL 30471*	Bacteria	HQ257512.1	Muraymycin	100%
NoTTA	WP_052373448	*Nocardia otitidiscaviarum*	Bacteria	JADLPU010000004.1	N/A	
KaTTA	WP_033354341	*Kitasatospora aureofaciens*	Bacteria	NZ_JNWR01000048.1	Valclavam	64%
PbTTA	MBN2478762.1	*Parachlamydiales bacterium*	Bacteria	JAFGQY010000010.1	N/A	
DbTTA	MBI5609283	*Deltaproteobacteria bacterium*	Bacteria	JACRCU010000288.1	N/A	

*Accession number for the obafluorin biosynthesis gene cluster

Table S3. Strains and Plasmids used in this study.

Number	Name	Relevant genotype	Source
E. coli strains			
	DH5α	F– Φ80*lac*ZΔM15 Δ(l*ac*ZYA-argF) U169 *rec*A1 *end*A1 *hsd*R17 (rK–, mK+) *pho*A *sup*E44 λ– *thi*-1 *gyr*A96 *rel*A1	NEB
	MG1655	F- λ- *ilv*G- *rfb-50 rph-1*	ATCC 700926
	MG1655 (DE3)	F- λ- *ilv*G- *rfb-50 rph-1* (λ DE3)<br>λ DE3 = λ sBamHIo ΔEcoRI-B int::(*lac*I::PlacUV5::T7 gene1) i21 Δ*nin*5	Previous study [2]
	RARE	MG1655(DE3) Δ*dkgB* Δ*yeaE* Δ*(yqhC-dkgA)* Δ*yahK* Δ*yjgB*	Previous study[2]
	BL21 (DE3)	*fhu*A2 [lon] *omp*T *gal* (λ DE3) [dcm] Δ*hsd*S	NEB
1-13	MAJ01-MAJ13	DH5α harboring TTA expression plasmids P1-P13	This study
14-26	MAJ14-MAJ26	BL21 (DE3) harboring TTA expression plasmids P1-P13	This study
27-39	MAJ27-MAJ39	MG1655 (DE3) harboring TTA expression plasmids P1-P13	This study
40-52	MAJ40-MAJ52	DH5α harboring SUMO-tagged TTA expression plasmids P14-P26	This study
53-65	MAJ53-MAJ65	BL21 (DE3) harboring SUMO-tagged TTA expression plasmids P14-P26	This study
66-78	MAJ66-MAJ78	MG1655 (DE3) harboring SUMO-tagged TTA expression plasmids P14-P26	This study
79-91	MAJ79-MAJ91	RARE harboring SUMO-tagged TTA expression plasmids P14-P26	This study
92	MAJ92	DH5α harboring TTA expression plasmid P27	This study
93-96	MAJ93-96	DH5α harboring CAR expression plasmids P28-P31	Previous studies[2,3]
97	MAJ97	RARE harboring pACYC-niCAR-sfp (P28) and pZE-SUMO-PbTTA(P25)	This study
98	MAJ98	RARE harboring pACYC-SUMO-PbTTA (P27)	This study
99	MAJ99	RARE harboring pZE-mavCAR-sfp (P29) and pACYC-SUMO-PbTTA (P27)	This study
100	MAJ100	RARE harboring pZE-mmCAR-sfp (P30) and pACYC-SUMO-PbTTA (P27)	This study
101	MAJ101	RARE harboring pZE-trCAR-sfp (P31) and pACYC-SUMO-PbTTA (P27)	This study
102-105	MAJ102-105	BL21 (DE3) harboring CAR expression plasmids P28-31	Previous study[3]
106-109	MAJ106-109	DH5α harboring ADH expression plasmids P32-P35	This study
110-113	MAJ110-113	BL21 (DE3) harboring ADH expression plasmids P32-35	This study
114	MAJ114	DH5α harboring PTDH expression plasmids P36. pET15b-17X-PTDH was a gift from Wilfred van der Donk (Addgene plasmid # 166786; http://n2t.net/addgene:166786 ; RRID:Addgene_166786).	Previous study[4]
115	MAJ115	BL21 (DE3) harboring PTDH expression plasmid P36	This study
Plasmids			
P1	pZE-ObiH	ColE1 ori, Kan^R, TetR, Tet promoter with a codon optimized *obiH* gene bearing an N-terminal hexahistidine tag.	This study
P2	pZE-PiTTA	ColE1 ori, Kan^R, TetR, Tet promoter with a codon optimized *piTTA* gene bearing an N-terminal hexahistidine tag.	This study
P3	pZE-BsTTA	ColE1 ori, Kan^R, TetR, Tet promoter with a codon optimized *bsTTA* gene bearing an N-terminal hexahistidine tag.	This study
P4	pZE-CsTTA	ColE1 ori, Kan^R, TetR, Tet promoter with a codon optimized *csTTA* gene bearing an N-terminal hexahistidine tag.	This study
P5	pZE-BuTTA	ColE1 ori, Kan^R, TetR, Tet promoter with a codon optimized *buTTA2* gene bearing an N-terminal hexahistidine tag.	This study
P6	pZE-StTTA-Δ36	ColE1 ori, Kan^R, TetR, Tet promoter with a codon optimized *stTTA-Δ36* gene bearing an N-terminal hexahistidine tag.	This study
P7	pZE-TmTTA	ColE1 ori, Kan^R, TetR, Tet promoter with a codon optimized *tmTTA* gene bearing an N-terminal hexahistidine tag.	This study
P8	pZE-RaTTA	ColE1 ori, Kan^R, TetR, Tet promoter with a codon optimized *raTTA* gene bearing an N-terminal hexahistidine tag.	This study
P9	pZE-SNTTA	ColE1 ori, Kan^R, TetR, Tet promoter with a codon optimized *snTTA* gene bearing an N-terminal hexahistidine tag.	This study
P10	pZE-NoTTA	ColE1 ori, Kan^R, TetR, Tet promoter with a codon optimized *noTTA* gene bearing an N-terminal hexahistidine tag.	This study
P11	pZE-KaTTA	ColE1 ori, Kan^R, TetR, Tet promoter with a codon optimized *kaTTA* gene bearing an N-terminal hexahistidine tag.	This study
P12	pZE-PbTTA	ColE1 ori, Kan^R, TetR, Tet promoter with a codon optimized *pbTTA* gene bearing an N-terminal hexahistidine tag.	This study
P13	pZE-DbTTA	ColE1 ori, Kan^R, TetR, Tet promoter with a codon optimized *dbTTA* gene bearing an N-terminal hexahistidine tag.	This study
P14	pZE-SUMO-ObiH	ColE1 ori, Kan^R, TetR, Tet promoter with a codon optimized *obiH* gene bearing an N-terminal hexahistidine tag followed by a SUMO tag and a TEV protease cleavage site.	This study
P15	pZE-SUMO-PiTTA	ColE1 ori, Kan^R, TetR, Tet promoter with a codon optimized *piTTA* gene bearing an N-terminal hexahistidine tag followed by a SUMO tag and a TEV protease cleavage site.	This study
P16	pZE-SUMO-BsTTA	ColE1 ori, Kan^R, TetR, Tet promoter with a codon optimized *bsTTA* gene bearing an N-terminal hexahistidine tag followed by a SUMO tag and a TEV protease cleavage site.	This study
P17	pZE-SUMO-CsTTA	ColE1 ori, Kan^R, TetR, Tet promoter with a codon optimized *csTTA* gene bearing an N-terminal hexahistidine tag followed by a SUMO tag and a TEV protease cleavage site.	This study

P18	pZE-SUMO-BuTTA	ColE1 ori, Kan^R, TetR, Tet promoter with a codon optimized *buTTA* gene bearing an N-terminal hexahistidine tag followed by a SUMO tag and a TEV protease cleavage site.	This study
P19	pZE-SUMO-StTTA-Δ36	ColE1 ori, Kan^R, TetR, Tet promoter with a codon optimized *stTTA-Δ36* gene bearing an N-terminal hexahistidine tag followed by a SUMO tag and a TEV protease cleavage site.	This study
P20	pZE-SUMO-TmTTA	ColE1 ori, Kan^R, TetR, Tet promoter with a codon optimized *tmTTA* gene bearing an N-terminal hexahistidine tag followed by a SUMO tag and a TEV protease cleavage site.	This study
P21	pZE-SUMO-RaTTA	ColE1 ori, Kan^R, TetR, Tet promoter with a codon optimized *raTTA* gene bearing an N-terminal hexahistidine tag followed by a SUMO tag and a TEV protease cleavage site.	This study
P22	pZE-SUMO-SNTTA	ColE1 ori, Kan^R, TetR, Tet promoter with a codon optimized *snTTA* gene bearing an N-terminal hexahistidine tag followed by a SUMO tag and a TEV protease cleavage site.	This study
P23	pZE-SUMO-NoTTA	ColE1 ori, Kan^R, TetR, Tet promoter with a codon optimized *noTTA* gene bearing an N-terminal hexahistidine tag followed by a SUMO tag and a TEV protease cleavage site.	This study
P24	pZE-SUMO-KaTTA	ColE1 ori, Kan^R, TetR, Tet promoter with a codon optimized *kaTTA* gene bearing an N-terminal hexahistidine tag followed by a SUMO tag and a TEV protease cleavage site.	This study
P25	pZE-SUMO-PbTTA	ColE1 ori, Kan^R, TetR, Tet promoter with a codon optimized *pbTTA* gene bearing an N-terminal hexahistidine tag followed by a SUMO tag and a TEV protease cleavage site.	This study
P26	pZE-SUMO-DbTTA	ColE1 ori, Kan^R, TetR, Tet promoter with a codon optimized *dbTTA* gene bearing an N-terminal hexahistidine tag followed by a SUMO tag and a TEV protease cleavage site.	This study
P27	pACYC-SUMO-PbTTA	P15A ori, Cm^R, *lac*I, T7*lac* with codon optimized SUMO-tagged PbTTA	This study
P28	pACYC-niCAR-sfp	pACYCDuet-1 harboring a codon optimized carboxylic acid reductase from *Norcardia iowensis* (niCAR) and a codon optimized phosphopantetheinyl transferase from *Bacillus subtilis* (sfp). P15A ori, Cm^R, *lac*I, T7*lac*	Previous study[2]
P29	pZE-mavCAR-sfp	ColE1 Ori, Kan^R, TetR, Tet promoter with a codon optimized carboxylic acid reductase from *Mycobacterium avium* (mavCAR) and a codon optimized phosphopantetheinyl transferase from *Bacillus subtilis* (sfp).	Previous study[3]
P30	pZE-mmCAR-sfp	ColE1 Ori, Kan^R, TetR, Tet promoter with a codon optimized carboxylic acid reductase from *Mycobacterium marinum* (mmCAR) and a codon optimized phosphopantetheinyl transferase from *Bacillus subtilis* (sfp).	Previous study[3]
P31	pZE-trCAR-sfp	ColE1 Ori, Kan^R, TetR, Tet promoter with a codon optimized carboxylic acid reductase from *Trichoderma reesei* (trCAR) and a codon optimized phosphopantetheinyl transferase from *Bacillus subtilis* (sfp).	Previous study[3]
P32	pZE-eutG-Ctermhis	ColE1 Ori, Kan^R, TetR, Tet promoter with an alcohol dehydrogenase (eutG) from *Escherichia coli.*	This study
P33	pZE-adhP-Ctermhis	ColE1 Ori, Kan^R, TetR, Tet promoter with an alcohol dehydrogenase (adhP) from *Escherichia coli.*	This study
P34	pZE-adhE-Ctermhis	ColE1 Ori, Kan^R, TetR, Tet promoter with an alcohol dehydrogenase (adhE) from *Escherichia coli.*	This study
P35	pZE-fucO-Ctermhis	ColE1 Ori, Kan^R, TetR, Tet promoter with an alcohol dehydrogenase (fucO) from *Escherichia coli.*	This study
P36	pET15b-17X-PTDH	pBR322 ori, AmpR, LacI, T7 promoter with a phosphite dehydrogenase (PTDH) from *Pseudomonas stutzeri* containg the following mutations for activity: A196R, T201S, A328T, E352N, C356D.	Previous study[4]

Table S4. Oligonucleotides used in this study.

All primers denoted FWD and REV were used for cloning whereas any primers containing SEQ were used for sequencing the associated plasmid.

Oligo Name	Sequence
pZE backbone FWD	CTTGATGGGGGATCCCATGGTA
pZE backbone REV	GTGGTGATGATGGTGATGGCTGCTGCCCATGGTACCTTTCTCCTCTTTAATGAATTCG
StTTA REV	CCATGGGATCCCCCATCAAGTTAACGAAAGACCTCACCCAACA
BuTTA REV	CCATGGGATCCCCCATCAAGTTAAGCGATTACTTCCTCCATCAA
PiTTA REV	CCATGGGATCCCCCATCAAGTTAGCGTTGAATTCCACGCTC
ObiH-REV	CCATGGGATCCCCCATCAAGTTAACGTTGGGCTCCTTGG
BsTTA REV	CCATGGGATCCCCCATCAAGTTAACGCATCACGCCTTGG
CsTTA REV	CCATGGGATCCCCCATCAAGTTAGCGTAACGCCTCCCCAATA
StTTA FWD	GCCATCACCATCATCACCACATGGGAGTTTGGGCAGGC
BuTTA FWD	GCCATCACCATCATCACCACATGATGACGGACTTCGCA
PiTTA FWD	GCCATCACCATCATCACCACATGAAACAAGACGAATCGAATG
ObiH-FWD	GCCATCACCATCATCACCACATGTCCAATGTCAAGCAACA
BsTTA FWD	GCCATCACCATCATCACCACATGAAACAGGAACCTACGGG
CsTTA FWD	GCCATCACCATCATCACCACATGACGCGCACGACCC
BsTTA SEQ	GTGCCCGAACATTCAGAG
StTTA SEQ	GCGTATATTGCGTTCCG
BuTTA SEQ	ACCATCCTGCGATGAAG
PiTTA SEQ	AAAGGGGTTTATTGCGTTCA
CsTTA SEQ	GCGGGTCATTTACATCGT
PiTTA SUMO FWD	GAAAATCTGTATTTTCAGGGCAAACAAGACGAATCGAATGTTG
TEV SUMO REV	GCCCTGAAAATACAGATTTTCTG
BsTTA SUMO FWD	GAAAATCTGTATTTTCAGGGCAAACAGGAACCTACGGGC
StTTA SUMO FWD	AAAATCTGTATTTTCAGGGCGGAGTTTGGGCAGGCGAC
pZE split REV V1	CCTGGTATCTTTATAGTCCTGTCGG
CsTTA SUMO FWD	AAAATCTGTATTTTCAGGGCACGCGCACGACCCCCCAG
pZE split REV V2	GGGAAACGCCTGGTATCTTTATAGTCCTGTCGG
ObiH SUMO FWD	AAAATCTGTATTTTCAGGGCTCCAATGTCAAGCAACAGAC
PbTTA SUMO FWD	AAAATCTGTATTTTCAGGGCGAAACCTCCCTGAAGGATTTTG
BuTTA SUMO FWD	AAAATCTGTATTTTCAGGGCACGGACTTCGCACAGGC
BuTTA SUMO REV	ACGCCTGGTATCTTTATAGTCCTGTC
RaTTA gene fwd	GCCATCACCATCATCACCACATGTTGGAAATTGTGGGGG
RaTTA gene rev	CCATGGGATCCCCCATCAAGTTAACGATAAAGCCACGCAG
pZE bbone fwd	CTTGATGGGGGATCCCATG
pZE bbone rev	GTGGTGATGATGGTGATGG
TmTTA gene fwd	GCCATCACCATCATCACCACATGCGCGAGGAAGAAGC
TmTTA gene rev	CCATGGGATCCCCCATCAAGTTACAGTAACGGAAGACAAGGG
SnTTA gene fwd	GCCATCACCATCATCACCACATGACATCAAGCGACGATTG
SnTTA gene rev	CCATGGGATCCCCCATCAAGTTACCCATGAAAAAGTCCCG
NoTTA gene fwd	GCCATCACCATCATCACCACATGAATACGTTCGATATCTTAGAAC
NoTTA gene rev	CCATGGGATCCCCCATCAAGTTATGCGACTGATACCTCC
PbTTA gene fwd	GCCATCACCATCATCACCACATGGAAACCTCCCTGAAGG
PbTTA gene rev	CCATGGGATCCCCCATCAAGTTAGAATAACTTCTCGTAGATCTCG
DbTTA gene fwd	GCCATCACCATCATCACCACTTGACGAATAATCGCGAGC
DbTTA gene rev	CCATGGGATCCCCCATCAAGTTAAGAGGCATAGACCGCC
KaTTA gene fwd	GCCATCACCATCATCACCACATGGATGTGTTGGCTGC
KaTTA gene rev	CCATGGGATCCCCCATCAAGTTAGGCTACTGCCAAGGG
SUMO tag fwd	ATGTCCCTGCAGGACTC
SUMO tag rev	GCCCTGAAAATACAGATTTTCTGAACCTCCACCTCCCGACCCACCACCGCCGCCACCAATCTGTTCGC
pZE-SWNB bbone rev	TCCGAGTCCTGCAGGGACATGTGGTGATGATGGTGATGG
pZE- TmTTA bbone fwd	AAAATCTGTATTTTCAGGGCATGCGCGAGGAAGAAGC
pZE- RaTTA bbone fwd	AAAATCTGTATTTTCAGGGCATGTTGGAAATTGTGGGGG
pZE- SnTTA bbone fwd	AAAATCTGTATTTTCAGGGCATGACATCAAGCGACGATTG
pZE- NoTTA bbone fwd	AAAATCTGTATTTTCAGGGCATGAATACGTTCGATATCTTAGAAC
pZE- TmTTA bbone rev	TCCGAGTCCTGCAGGGACATGTGGTGATGATGGTGATGGC
pZE- DbTTA bbone fwd	AAAATCTGTATTTTCAGGGCTTGACGAATAATCGCGAGC
pZE- KaTTA bbone fwd	AAAATCTGTATTTTCAGGGCATGGATGTGTTGGCTGC
pACYC bbone fwd	AAGCTTGATGGGGGATC
pACYC bbone rev	GGTATATCTCCTTATTAAAGTTAAAC
pACYC SUMO-PbTTA12 ins fwd	CTTTAATAAGGAGATATACCATGGGCAGCAGCCATCA
pACYC SUMO-PbTTA12 ins rev	GGATCCCCCATCAAGCTTTTAGAATAACTTCTCGTAGATCTCGT
pZE-cterm-his bbone fwd	GGCAGCAGCCATCACCATC
pZE-cterm-his bbone rev	GGTACCTTTCTCCTCTTTAATGAA
pZE eutG fwd	TTAAAGAGGAGAAAGGTACCATGCAAAATGAATTGCAGACC
pZE eutG rev	TGATGGTGATGGCTGCTGCCTTGCGCCGCTGCGTACAG
pZE adhP fwd	TTAAAGAGGAGAAAGGTACCATGAAGGCTGCAGTTGTTAC

pZE adhP rev	TGATGGTGATGGCTGCTGCCGTGACGGAAATCAATCACCA
pZE adhE fwd	TTAAAGAGGAGAAAGGTACCATGGCTGTTACTAATGTCGC
pZE adhE rev	TGATGGTGATGGCTGCTGCCAGCGGATTTTTTCGCTTTT
pZE fucO fwd	TTAAAGAGGAGAAAGGTACCATGGCTAACAGAATGATTCTG
pZE fucO rev	TGATGGTGATGGCTGCTGCCCCAGGCGGTATGGTAAAG

Table S5. DNA G-Blocks/Twist gene fragments for cloning in this study. Start codons for each gene are underlined.

Oligo Name	Protein Accession Number	Sequence
**ObiH**	ARJ35753.1	ATGTCCAATGTCAAGCAACAGACAGCTCAGATCGTGGATTGGTTATCAAGCACTTTAGGTAAAGACCATCAG<br>TATCGTGAAGATAGCTTGAGTCTTACAGCGAACGAGAACTATCCGTCAGCGTTGGTACGTTTGACGTCGGG<br>CTCGACCGCAGGGGCGTTTTATCACTGTAGTTTCCCCTTTGAGGTACCTGCCGGGGAATGGCACTTCCCGG<br>AGCCAGGGCATATGAATGCCATCGCAGACCAGGTACGTGATCTTGGGAAAACACTGATCGGAGCACAGGC<br>GTTTGACTGGCGCCCAAACGGCGGCTCTACAGCAGAACAGGCGTTGATGTTAGCGGCGTGCAAGCCCGGG<br>GAAGGATTTGTCCATTTCGCACACCGCGACGGAGGCCATTTTGCGCTTGAATCACTGGCGCAGAAAATGGG<br>AATTGAAATTTTCCACTTGCCAGTTAACCCCACGAGTTTGCTTATTGATGTGGCGAAATTGGATGAAATGGTC<br>CGCCGCAATCCGCACATCCGTATTGTAATTCTGGACCAGTCCTTTAAGCTTCGCTGGCAGCCGTTGGCGGA<br>AATTCGTTCCGTACTGCCGGATTCGTGTACTTTGACGTACGACATGAGTCACGATGGAGGTTTGATTATGGG<br>TGGCGTTTTCGATTCGCCTTTAAGTTGCGGAGCAGACATCGTACACGGAAACACACATAAGACGATCCCTG<br>GTCCACAGAAAGGGTACATCGGATTTAAGAGTGCTCAACACCCGCTGTTAGTGGATACCAGCCTTTGGGTA<br>TGCCCTCACCTGCAATCCAACTGCCATGCGGAACAGCTGCCGCCAATGTGGGTAGCATTCAAAGAAATGGA<br>ACTGTTCGGGCGTGATTACGCGGCCCAAATTGTGTCAAATGCTAAGACCTTGGCACGTCACTTGCACGAGT<br>TAGGATTAGACGTTACGGGGGAGAGCTTTGGGTTTACCCAGACTCACCAGGTACACTTCGCTGTAGGCGAC<br>TTACAAAAAGCCTTGGATTTATGTGTTAATTCACTTCACGCAGGGGGCATCCGTAGCACGAATATCGAGATT<br>CCCGGAAAACCAGGGGTGCATGGTATTCGTTTGGGTGTGCAAGCGATGACTCGCCGTGGCATGAAGGAAA<br>AGGATTTCGAGGTGGTAGCTCGTTTCATTGCGGATCTTTACTTCAAGAAAACTGAGCCAGCGAAAGTTGCTC<br>AGCAGATTAAGGAATTTTTGCAGGCGTTCCCATTAGCGCCTCTGGCATATTCTTTTGATAATTATTTAGACGA<br>GGAGTTATTGGCTGCGGTGTACCAAGGAGCCCAACGTTAA
**PiTTA**	WP_095149064.1	ATGAAACAAGACGAATCGAATGTTGGTCCTGTCATTGACTGGCTGGCTCAGACCCTTGGACAGGACTACAA<br>GTACCGCCAGGACACACTTTCACTTACAGCTAACGAAAACTACCCTTCAGAGCTTGTTCGTCTGACCAGCG<br>GCTCTACAGCCGGGGCATTTTATCACTGCTCTTTTCCGTTCCCCGTTCCTCTTGGAGAATGGCATTTCCCAG<br>AGCCAGGACAAATGAACGAGATCGCCGATGATCTGCGCGGTTTGGCCAAACGTATGATGGGTGCGCAGGC<br>ATTCGATTGGCGCCCTAATGGTGGGAGCCCGGCTGAACAGGCCTTGATGTTAGCGGCTTGTAAACAAGGT<br>GAAGGTTTTGTACACTTTGCACATCGCGATGGGGGGCATTTTGCTTTAGAGCAATTGGCGACAAAAATGGG<br>TATTGAGATTTTCCATTTACCTGTGGATCCGCAAAGTCTGCTTATTGACGTTGCTAAGCTTGATGACATGGTC<br>CGCCGTAACCCTCACATCCGTATCGTAATTCTTGATCAATCCTTCAAACTTCGTTGGCAGCCGTTAGCCGAG<br>ATTCGTGCAATCCTTCCCGATTCATGCACTTTAACTTATGATATGTCTCATGATGGGGGCCTTATTCTGGGTG<br>GGGTCTTCGATAGCCCATTGGCGTGCGGTGCGGATATCGCTCACGGCAATACTCACAAGACTATTCCGGG<br>GCCTCAAAAGGGGTTTATTGCGTTCAAGAGCGCTCAGCACCCCCTGTTGGTGGAAACCAGTCTTTGGGTAT<br>GTCCACACTTACAGAGTAACTGTCACGCCGAACTTTTACCCTCTATGTGGGCCGCATTCAAGGAGATGGAA<br>GCTTTTGGCCCCGCCTATGCCCACCAGATGGTGCGCAATGCTAAGGCGTTGGCCAACCAACTTCACGAGCT<br>TGGTTTAAATGTTTCGGGAGAGTCTTTTGGATTTACAGAGACGCACCAGGTGCATTTCGCCGTAGGAGATTT<br>ACAACAGGCGTTGAGTATGTGCGTGGACTCGTTACACGCGGGCGGAATCCGCTCGACTAACATCGAGATC<br>CCGGGAAAGCCCGGGATGCACGGGATCCGCTTGGGGGTACAGGCCATGACCCGCCGCGGTATGAAAGAG<br>GATGACTTTCGTCGCGTCGCTGGCCTTATCGCTGACCTTTACTTCAAGCGTACCGAACCTGCACGTGTTGC<br>TTCAAAGGTGAAGGAGTTATTGGGCGATTTTCCACTTGCCCCTCTGGCCTACTCGTTCGATCAACAAATCGA<br>CGAGTCTCGCCGCCGTTTGCTTGAGCGTGGAATTCAACGCTAA
**BsTTA**	WP_060149112.1	ATGAAACAGGAACCTACGGGCGCCTTCGAGGTTGCCACGGTGCTGAACGACATTTTTCTTGCTGACCATCG<br>CTACCGCGAGGTAACTCTTAGTCTTACCGCTAATGAAAATTATCCTTCAGAGCTTGTACGTGTTACGTCCGG<br>AAGTACCGCCGGGGCTTTTTATCATGTGAGCTTCCCGTTCGATGTACCCGATGGAGAATGGCACTTCCCCG<br>AACCCGGACATATGCACGCGGTGGCGGATAAAGTTCGTAGTTTGGGGAAGTCATTGCTGCATGCACAGACA<br>TTTGATTGGCGTCCAAACGGTGGCTCTGCGGCGGAACAGGCGTTAATGCTTGCGGCCTGTCAACCCGGTG<br>ATGGTTTCGTTCATTTCGCACATGGAGACGGAGGGCACTTCGCCTTAGAGGCTCTGGCATCAAAAGCAGGT<br>ATCGAAATCTTTCATCTGCCAGTTGACCCAGACACGCTGCTTATTGATGTGAATCGTTTAGCTACGTTAGTG<br>GACGCACATCCACGTATTCGTATTGTCATTTTGGACCAGTCATTTAAACTTCGCTGGCAGCCTCTGCGCGCG<br>ATCCGTGATGCACTTCCTGCACATTGTACGTTGACTTACGATGCTAGCCACGATGGAGGGCTGGTTATGGG<br>AGGATGGTTTGACAGCCCGCTTCGTTGTGGTGCTGACGTAGTTCATGGTAATACCCATAAAACTATTGCAGG<br>GCCTCAGAAAGCTTATGTTGCTTTTGGCTCTGCTGAGCACCCCTTATTAGCAGATACCAGTATTTGGGTGTG<br>CCCGAACATTCAGAGCAATTGTCATGCAGAACAGCTGCCATCTATTTGGGTTGCATTGAAAGAAATCGAAGC<br>ATACGGGCCTGCATATGCGTCCCAGGTAGTGCGTAACGCGACAGCGTTTGCTCGTGCTTTACACGCGCGT<br>GGGCTTGACGTGTCAGGAGAGTCCTTTGGGTTCACCGAAACCCATCAAGTCCACTTCAGCGTCGGGACCC<br>CGGAGGCAGCGTTATTGACATGTCGTGACGTGTTGCACCGCGGGGGAATCCGTACCACGAACATCGAGCT<br>TCCGGGTAAGCCGGGGGTACATGGCATCCGTCTTGGAGTACAGGCAATGACGCGTCGTGGAATGGTCGAG<br>CGCGACTTTGAAACCGTCGCCGACTTTATCGCTGCGCTTTGTACACGCAAACGTACACCCGAGGATGTGGC<br>TCCGGATGTCGAAACGTTCCTGGGTGACTTCCCATTATCCCCACTTGCATTTTCCTTCGACGGGGGTATGAC<br>TGACGCATTGCGTGCCGCACTGCGCCAAGGCGTGATGCGTTAA
**CsTTA**	WP_018749561.1	ATGACGCGCACGACCCCCCAGGCACGTCATGTCGTGGAGCGCCTGAATTCAGTTTTAGGACAAGACTACC<br>GCTATCGTGAGGATTGTCTGAGCCTTACCGCGAATGAGAACTATCCTTCCGCATTAGTGCGCTTAGCGGGG<br>AGTGCCACAGCTGGAGCCTTCTACCACTGTAGCTTTCCGTTTGAGGTGCCACCGGGAGAATGGTATTTTCC<br>TGAGAGCGGTCGTATGGGGGAACTTGCTCAACAGCTGAATGAATTAGGTCGTTCGTTATTAGGCGCGGGTA<br>CATTCGATTGGCGCCCCAACGGTGGCTCGCCAGCGGAGCAGGCATTGATGTTAGCGGCCTGCAAGCACGG<br>TGAAGGGATGGTCCATTTTGCTCATCGTGACGGTGGCCACTTTGCGCTGGAGAATCTGGCGCAAAAAGCTG<br>GTATCGACATCTTTCATTTGCCTGTAGATCCCCAGACGTTGTTGATCGATGTTGCACGCCTTGACGAGCTTG<br>TCCGCCGCAATCCTCAAATCCGTATTGTGATCTTGGACCAGTCTTTTAAGTTACGCTGGCAACCCCTTGCAG<br>CGATCCGCAAGGTTCTTCCCCCATCGTGTACACTTACCTATGACACCTCTCATGATGGTGGACTTATTATGG<br>GAGGAGTTTTTGATTCTCCCTTGCATTGTGGTGCAGACGTAATTCATGGCAACACGCATAAAACAGTGCCCG<br>GACCGCAGAAGGGGTATATCGCCTTCAAATCCGCTGAGCATCCTTTGTTGGTTGACACGAGTCTGTGGCTT<br>TGCCCACATTTGCAGTCTAACTGTCATGCCGAGCTTTTGCCTCCAATGTGGGTGGCTTTTAAAGAAATGGAG<br>GCTTTCGGACATGATTACGCCCCTCAAGTGGCCCGCAACGCGAAGGCTCTGGCGGGTCATTTACATCGTTT<br>AGGATTCGAGGTTTCAGGCGAGGCTTTCGGTTTCACTGAAACCCACCAAGTGCATTTTGCCGTAGGAGACT<br>TGCAGCAAGCGCTTGATTTGTGCATGAACACCTTGCATCGTGGGGGCATCCGCTCTACGAATATTGAAATC<br>CCGGGTAAACCCGGCATTCAGGGTATTCGCCTGGGCGTTCAGGCTATGACCCGTCGCGGTCTGCGCGAAG

		ATGATTTTGAGCAGGTGGCGCGTTTTATCGCGGACTTGCACTTCCGCAAAGCAGACCCAGCCGGAGTCGCA<br>GCACAAGTAGCGGAATTTCTTCGTGCTTTTCCTTTGGCACCATTACATTACTCATTTGATCAGGAACTGGATC<br>ATGAGTTATTGCAGTCCCTTATTGGGGAGGCGTTACGCTAA
**BuTTA**	WP_080410754.1	ATGATGACGGACTTCGCACAGGCGGTAGTAAACCCGTTCGTAGATGAGCAGCGTAAGTCCCGTTTAGTAGA<br>AAAAATCTCAAACATCTTCGATAGTCTTCATAGCGATTTTGCCTTGGATAATTTATACCGCGCAAGCCACTTA<br>AGTCTGACCGCCTCTGAGAATTATCCATCCCGCTTTGTGCGCACGCTGGGAGCCGGTATGCAAGGCGGTTT<br>CTATGAATTCGCGCCACCTTACGCCGCTAACCCAGGAGAGTGGTACTTCCCTGACAGTGGCGCGCAGTCG<br>AGTCTGGTCGAGAAACTTGCTAGTTTGGGAAAACAGTTGTTCGAGGCTAACTCGTTTGACTGGCGTCCCAA<br>CGGGGGATCAGCAGCGGAACAGGCTGTGCTTTTAGGCACATGTGCCCGCGGGGATGGCTTCGTCCACTTT<br>GCTCACAAGGATGGCGGCCACTTTGCTCTGGAAGAGTTGGCCCAGAAGGTGGGAGTTAGCATCTTCCATCT<br>GCCAATCGAGGAGAAGAGTCTTTTGATTGATGTTGACCGCCTGGCGACATTAATCAAAGATAACCCCCACAT<br>TAAGCTTGTAATTCTGGACCAATCGTTTAAGCTTCGCTGGCAACCTTTACTGCAAATCCGCCAAGCCTTACC<br>GGAATCAGTCGTATTATCGTACGACGCGAGTCACGACGGGGGATTAATCATCGGCGAATGCCTGCCCCAG<br>CCATTACTTTTCGGAGCGGATATTGTTCACGGGAATACACACAAGACAATTCCGGGCCCGCAAAAGGGTTA<br>CATTGCGTTCAAGAATGTAGACCATCCTGCGATGAAGCATGTTAGCGATTGGGTTTGTCCTCATTTGCAATC<br>TAACTCGCATGCCGAGTTGATCGCACCCATGTATATTGCCTTGGTTGAAATGTCTTTGTACGGACGCAGTTA<br>CGCGGAGCAGGTTATTAAAAATGCTAAGGCGTTGGCACACGCCCTGCACGCCGAGGGAGTACGCGTCTCG<br>GGCGAATCGTTCGGTTTTACAGAAACACACCAAGTTCATGTTGTTGTTGGGTCCGAGCGTAAAGCGTTGGA<br>GTTAGTTACTGGTACCTTGGCATTGGCAGGAATTCGCTGCAACAACATCGAGATTCCAGGCGCGAACGGCT<br>TATTTGGTTTGCGCTTAGGAGTGCAGGCATTGACGCGTCGCGGAATTAAAGAGCACGGGATGGCTGAAGTT<br>GCCCGTTTTTTAGTGCGCTTGATTCTGAAAAACGAATCCCCCACGGCCATCCGCAACGAAATTGCGTCATTT<br>CTTGAATCATATCCTATTAATACGCTTCATTATTCATTAGATGCTCACTATTATACCCCTTCGGGTATTAAATT<br>GATGGAGGAAGTAATCGCTTAA
**StTTA-Δ36**	WP_101279775.1	ATGGGAGTTTGGGCAGGCGACCGTGTTGCCCAAGTTTTGGAACGCTTAGCGTCGGATTTTGTTTTAGACAA<br>CACTTATCGCGAACAACACCTGAGCTTGACGGCTTCTGAGAACTATCCTTCAAAACTGGTACGCATGTTGGG<br>AGCGGGATTACAGGGGGGTTTCTATGAGTTTGCTCCGCCCTATCCGGCAGAAGCAGGAGAATGGGCATTC<br>CCGGACTCCGGAGCGAACGCGTCCCTTGTAGGGAAGCTGACTGGCATTGGTCGCCAACTGTTCGAAGCCG<br>CAACATTCGACTGGCGTCCGAACGGCGGATCCGTGGCCGAGCAAGCAGTATTGCTGGGGACGTGTGGAC<br>GCGGGGATGGTTTTGTGCACTTCGCGCATAAGGATGGGGGCCACTTTGCGTTGGAGAGTCTGGCGGGTGC<br>TGCCGGAGTCAACACGTATCATCTGCCCATGGTAGACCGCACGCTTCTGATCGATGTCGATCGTTTGGCTA<br>CTTTATGCGCTGAACACCCGGAAATTAAGTTAGTAATCTTAGATCAGTCCTTCAAATTACGCTGGCAACCGC<br>TTGCTCAAATCCGCGCCGCGCTGCCCGAGGGCGTATTTTTAGCTTATGACGCGTCTCATGACGGTGCTTTG<br>ATTGCTGGGGGTGTTCTGCCACAGCCTACCCTGTTAGGGGCCGATGCAGTTCATGGCAACACGCACAAAAC<br>GATCGCGGGGCCTCAAAAGGCGTATATTGCGTTCCGCGACGCTGAGCACCCCAAGTTACGTGCCGTCAGT<br>GATTGGGTGTGTCCACAGATGCAGAGTAATTCACATGCGGAACTGATCGCACCCATGTATGTAGCACTGTC<br>GGAGGTCGCCTTATATGGTCATGCGTATGCCCGCCAAATCTTAGCAAACGCCCAAGCGTTAGCGCACGGAT<br>TACACGAAGAGGGGGTCCGCGTATCTGGAGAGTCCTTCGGCTTTACAGAAACTCATCAAGTACACGTCGTG<br>ACGGGTTCAGCTGCGGATGCTCTGCGCCTGTCCTTGGGTGAGCTGGCCCAGGCAGGAATCCGTACGACAA<br>ACATTGAGGTACCAGGGGCAAATGGACTGCATGGTTTGCGCTTAGGAGTTCAAGCTATGACTCGCCGTGGT<br>TTACGCGAGCCACAGATGCGTGAAGTGGCACGCTTGGTTGCCAAAGTTGTTTTGCGCCGTGCCGAACCAG<br>CGGCTGTACGCGCGGAGGTTGCGGATTTGTTACAGCATCACCCGTTAGATCAGTTGGCGTATTCCTTCGAT<br>TCCTACGTTGACTCGCCAGCTGCGGCGCGTTTGTTGGGTGAGGTCTTTCGTTAA
**TmTTA**	WP_188596100	CCATCACCATCATCACCACATGCGCGAGGAAGAAGCGATTGCGGCGCTGTCAAAATTACGCGCAATCATGG<br>ACCGCCATAACAACTGGCGCCGCCGTGAGACAATTAACTTAATTCCAAGCGAAAACGTGATGTCGCCGTTA<br>GCCGAGTATTTCTACTTAAATGATATGATGGGACGTTATGCTGAAGGAACGATTGGTAAACGCTACTACCAA<br>GGTGTATCGCTGGTGGACGAGGCGGAACAAATGTTAGTCGATTTAATGAGCTCTTTGTTTTCCTCGCGCTTT<br>ACAGACGTCCGCCCCATCAGCGGTACAGTTGCCAATATGGCCGTGTATCACTCAGTCGCGGGGCTTGGGG<br>AGAAGATCGCCTCTTTACCAACAGCCGCCGGGGGCCATATTTCGCATAACGAGACTGGTGCCCCCAAAGCA<br>TTCGGATTACGTGTTTCATATTTGCCGTGGTCTCAGGAAAACTTTAACGTGGATGTGGACGCTGCGCGTCG<br>CTTAATTGCCGAAGAACGCCCAAAATTGGTGTTGCTTGGGGCGTCACTTTATTTATTTCCTCATCCCATTAAA<br>GAATTAGCGGACGCTGCTCACGAGGTAGGTGCGGTTCTGATGCATGACTCAGCTCACGTACTTGGTTTAAT<br>TGCTGGTCATCAGTTCCCTAATCCTCTTGAACTTGGGGCGGACATTATGACTAGCAGCACGCACAAAACTTT<br>TCCGGGACCCCAAGGCGGTGTGATTTTTACCACACGTGAAGATTTGTTCAAGGAGATCCAACGCTCAGTTTT<br>CCCAGTAATGACATCGAATTATCACTTGCATCGCTATGCCTCGACGATTGTGACAGCTATTGAGATGAGTAC<br>GTATGGAGACGAATATGCAGCTACAGTGCGCTCCAACGCGAAAGCACTGGCGGAACAACTTCATGCCAAC<br>GGTTTACCTGTAGTTGCCGAAGAACACGGCTTCACGGCTACCCACCAGGTGGCAATGGATGTTTCAAAATT<br>TGGAGGCGGGGGGCCAATCGCTAAAGCGTTGGAGGACGCGAATATTATTGTAAACAAGAACATGCTGCCC<br>TGGGATAAGTCTCCGGTCAAACCATCCGGTATTCGCATGGGAGTTCAAGAAATGACTCGCATGGGAATGGG<br>TAAAGGCGAGATGGCGGCCGTGGCGGAGCTGATCGCAAAGGTGGTCATCAAAGGGGTCGAACCGTCTAAA<br>GTAAAGCCAGAGGTCGTCGAGTTGCGCCGCGGTTTCACAAAGGTACGCTATGGTTTTGATTTATCTACTTTG<br>GGCTTGAATTGCCCTTGTCTTCCGTTACTGTAACTTGATGGGGGATCCCATG
**RaTTA**	GIH11859	CCATCACCATCATCACCACATGTTGGAAATTGTGGGGGACCATGAACGCAAAATGGCGAGTGCAGTGAATC<br>TTATCCCCAGCGAGAATTTATTAACACCCGCCGCACGTTTAGCCTACCTTTCAGATGCGTATTCGCGTTATTT<br>TTTCGATGAGCGTGAGGTGTTCGGAAAGTGGTCTTTCCAAGGGGGGAGCATTGTGGGCGAAGTACAACGT<br>GAGGTTTTAGTGCCTCTGGTACAAAAGGTAACTGGGGCACGCCATGTGGACGTCCGTGGGATTAGTGGCC<br>TGAATGCCATGACCGTGGCTCTGGCAGCGTTTGGCGCCCGTGACCGCGTTACAATTACAGTACCGCCCCG<br>CCACGGAGGCCATCCAGCTACCGCAGTTGTGGCCGGACACTTTGGGCATCGTGCAGAGGCTTTACCTTTC<br>CGTGATGAAGCCTGGTGGGAGGTTGACTTGCCTGCCTTAGCGGAGTTAGTAGCTCGTACTGATCCGGCGTT<br>AGTTTATGTAGATCAGGCCACCGCTCTGGTCCCACTGGATTTAGCCGGAGTAATCCGCACCGTCAAGGAAG<br>TTTCCCCTGGGACACACGTACACGCCGACACATCGCACATCAACGCGTTCGTTTGGTCGGGATTGTTCGGC<br>CAACCACTTGACTTGGGGGCGGACAGTTACGGAGGCTCCACGCATAAGACCTTTGCGGGCCCTCATAAGG<br>CTTTATTGCTTACTAACGATGACGCAGTGAGCGATAAACTGACCTCCGTCGCAGTGAATCTTGTTTCGCATC<br>ATCATGTCAGCGACGTTGTAGCTTTAGCTATCGCCATGGTAGAGTTCGCGGAATGTGGCGGGGTAGATTAC<br>GCGCAGGCAGTTTTAGCAAATGCAGCGGCGTTCGCCCGCGCCCTGGCCGATGCCGGGCCTGGCGTACAA<br>GACGCGGGTGGTGTCTTAACCCGTACGCATCAAGTATGGTACGAACCTGCTGGCGATCCGCACCGCATTA<br>GCGAGCGCTTGTTCGATGCGGGGATCGTTGTGAACCCTTACAACCCTCTGCCGAGTACCGGTCGTTTAGGA<br>ATCCGTATGGGGTTAAATGAGGCGACCAAGTTAGGATTCGGAGAACCGGAAATGGCCGAGTTAGCAGGGT<br>TGCTTCACGGTGTAGCGGTTGACCGTATCGCCGTGGCTGAGGCGGGAGAGCGTGTGGCTGCCATGCGTCA<br>AGCCGCTCGTCCCGCGTATTGTTTTTCTGAAGATGTGGTCGCCTCTAAGCTTCGCGAGCTTACCGGAGCCT<br>CAGGTGCAGGTGTGGATGAGTTGGCTGCGTGGCTTTATCGTTAACTTGATGGGGGATCCCATG

**SnTTA**	ADZ45329	CCATCACCATCATCACCACATGACATCAAGCGACGATTGTGCTGCGAGTCGTACGGCTCCCGTCGCTGGCC<br>GCGCAGAACTTTTGGCGCTGTTGGGAGAAATCGAGAAGGAGCAGCGCATCAACGAGGCCGCCGTGAACTT<br>AGTGCCTTCAGAGAATCGCATTAGTCCCTGGGCTGGGGCGCCGTTACGTACCGATTTTTACAACCGCTATT<br>TCTTCAACGATTCTCTGGACCCCCAGGGATGGCAATTTCGTGGAGGGGAAGGGATTGGACGCCTGGAAAA<br>GGAGTTGGCTCTGCCCGCTTTACGCCGTTTAGGGCGTGCCGATCACGTTAACATCCGTCCTGTGTCAGGTA<br>TGAGTGCCATGCTTGTGGTCCTTTTAGGTTTGGGAGGCGAACCTGGGGATGGTGTAGTGTGTGTAGACGCA<br>GAAACGGGAGGTCATTATGCTACTGGCCGCCAAATCGCAATGTTAGGCCGCCGCCCTTTGCCCGTCCGCG<br>TGGTAGCGGGACGCGTTGATTTGGATGCTCTTCGCACGGCATTAACTAGCTGCCACGTTCCCTTGGTATAT<br>CTTGACCTTCAGAATTCACTTTGGGAGCTTGATGTTGCGGGAGTAGCCGAGGTCATCGCACGTACAAGCCC<br>ACGTACTGTTCTGCACGTGGACTGCAGCCACACATTAGGATTAATCCTTGGGGGCTCACATAAAAATCCATT<br>AGACTTGGGTGCGGATACGACTGGGGGGTCGACCCATAAAACTTTCCCAGGTCCGCAGAAAGGGGTTTTG<br>TTCACACGTGACGAGAACTTGAGTCGTAAGATCCGTGATGCTCAATTTTTCACGATCAGTTCACATCACTTC<br>GCGGAAACACTGGCGTTGGCCTTAGCGGCTGCAGAATTTGAGCATTTTGGCGCAGCCTATAGCCGCCAAG<br>TCCTTATCAATGCTCGCGCTTTTGCACACCGCTTACGCGAGCGCGGATTTGGAGTCGTTGAAGGCGGCCCG<br>CAGCTGACGGATACTCACCAAGTCTGGGTCCGCTTACCTCTTGAAGAATCGGCAGATGCCTTTAGCGCTCA<br>ATTGGCGTCCTTAGGTATCCGCGTCAATGTCCAGACTGAGTTGCCAGACATCCCTGAACCAGCCCTGCGCT<br>TAGGCGTGAGCGAGATTACTCTTAATGGTGGACGTGAGCCAGCAATGGAAACGTTGGCAGAGATCTTCGCT<br>TTGGTACGCGCAGGGGAGGCGACTAAGGCTGTCGATTTATTCCAAGTTCTTCCCCATGAAATGGGGGAACC<br>GTATTTTTTTACGGGATTACCTCAAGAAGCGGGACTTTTTCATGGGTAACTTGATGGGGGATCCCATG
**NoTTA**	WP_052373448	CCATCACCATCATCACCACATGAATACGTTCGATATCTTAGAACAACTTGCACGTTATGAGGTAGGCACATC<br>GCGCCGTTTGCATTTAATTGCGTCTGAGAATCCCCTGGACTCAGACACACGTGTGCCGTATATGCTTGCAG<br>GAACTTTAGCTCGTTACGCATTTGGGGAGCCGGGTCAGCCCAACTGGGCTTGGCCAGGCCGTGAGACTCT<br>GATTGACCTGGAAGCTGACACTGCGGCAGCCCTTGGGGCTTTGCTGGGCGCCGATCATGTTAATCTTCGTC<br>CGACTAGTGGTCTTTCAGCTATGACCGTGGCCTTGTCCGCCTTGGCCGAACATGCTGGGGACCGTGCAACT<br>GTTTTATCGCTTGCAGAATCAGATGGTGGCCATGGATCGACGGGGTTCATGGCCCGTCGTTTTGGGCTGGA<br>CTGGCAACGCATGCCCGCTGACCCGCGTACAGGCGTTGTGGATCTGGACGCACTGGCGCGTCAGGCTCG<br>CAGTGCCCGCGGTCCTCTGGTCTTATATCTGGATGCGTTCATGGCGCGCTTTCCTTTTGACTTAACGGGTAT<br>CCGCGGTGCGGTGGGTGACTCAGCTTTGATCCATTACGACGGTTCACATCCTTTGGGATTAATCGCGGGAG<br>GCCGTTTCCAAAATCCGTTAGCTGAAGGCGCCGATTCGCTTGGAGGGTCTGTACACAAAACCTGGCCTGGA<br>CCGGTAGGGAAAGGGATCATCGCTACCAATGATAGTGCACTTGCATCTCGCTTCGATACTCACGCCGCGGG<br>TTGGATCTCCCACCATCACCCTGCGGATCTGGCTGCACTGGCGCTTAGTACCGCCTGGATGGAGCAACATG<br>CTGGCGACTACGCGACAGCAGTGATCGCAAATGCCGTGCAATTAGCTGATGAACTTGCAGACGGCGGCTT<br>GAGCATCTGTGCCGATGACCGTGGTGCTACGGCGAGTCATCAAGTGTGGGTTGATATTGCTCCTATCTGTC<br>CAGCTCCTGTCGCGGCTCAGCGTTTGTATGATGCTGGTATTGTGGTAAACGCGATTGCAATCCCAGGGCTT<br>GCCGAACCCGGCTTGCGCCTGGGCGTTCAGGAGTTGACTCGCTGGGGATTAGACCGTGATGGAATGACAG<br>TCCTGACCTGGGTACTGACCCAACTGCTGGTCCATAACGCGGCCACAGCAGTGGTGGCCCCGCAAATGGA<br>AGCGTTGCGTACCGGCCTGACGCTGCCTGAAGATCGTCATGGGCTGGAGGGTTTTCTTCGTGCGTGTGAT<br>CCACAGGAGGTATCAGTCGCATAACTTGATGGGGGATCCCATG
**KaTTA**	WP_033354341	CCATCACCATCATCACCACATGGATGTGTTGGCTGCCCTGGAACGTAAGCACAGTTTAAACTTGTTTCCGAT<br>TGAAAATCGCTTGTCACCCCGTGCTGCCGCCGCTCTGGCATCCGATGCCGTAAACCGTTATCCGTACAGTG<br>AGACGGATGTGGCGGTGTACGGAGACGTTAGTGATCTGAATGCTGTATATGACCATTGCGTCAGTCTTACC<br>AAGGAATTTTATGGCGCCCGTCATGCATATGTTCAGTTTCTTTCCGGACTTCACACCATGCATACAGTGTTAA<br>CAGCAGTCACACCGCCAGGGGGCCGTGTAATGGTCATTGCGCCTGAAGACGGAGGACATTATGCAACGGT<br>TACTATTTGCCAAGGTTTTGGCTACCGCGTAGAGTACGTACCATTCGATCGCCAGACTTTGGAAATTGACTA<br>CACTGCTCTTGCCGAACGCACAGCCGAACATCCGGCTGATGTGATCTACTTGGACGCATCGACGGTATTGC<br>GCATGCCTGACGCGCGCTCTGCGTGCAGCAGCCCCAGGCGCTGTTCTGTGTCTGGATGCAAGTCATCT<br>TCTGGGACTTCTTCCCGCAGCCCCTGGGACCTTGGTCCTTGATGCTGGCTTTGATTCAATTTCTGGAAGCA<br>CTCACAAAACTTTACCGGGACCCCAAAAGGGATTGTTGGTGACAAACTCCGATGCCATTGCCGAACAGGTC<br>GGAGCGCGCATCCCTTTTACCGCGAGTTCATCGCATTCTGCGAGCGTGGGTTCGCTGGCGATTACATTAGA<br>AGAGCTTTTGCCCCATCGCGGGGATTACGCACGTCAGGTGATCGCAAACGCCCGTGAGCTGGCTCGTCAA<br>CTTGCGGCCCGCGGCTTTGACGTGGCAGGGGAAGCCTTCGGATTTACTGATACTCATCAGGTGTGGGTCC<br>ACCATCCAGAGGGAAATACACCGCATGAGTGGGGACGTCTGCTGACAGCTACTGATATTCGCACCACTACA<br>GTAGTGCTTCCATCAACTGCACGTAGTGGATTACGTTTAGGAACGCAGGAGTTGACACGTTGGGGGATGAA<br>GGAAGACGATATGACTACCGTTGCAGAGCTTCTTGCCCGTCTGCTTTTACGCGGAGAACAGAGTCGCTCAG<br>TTGCCGCGGATGTACGCGACTTGGCTCGTTCGTTCCCAGGTGTGGCTTTCGCGGACCGTCCAGCACCCTT<br>GGCAGTAGCCTAACTTGATGGGGGATCCCATG
**PbTTA**	MBN2478762.1	CCATCACCATCATCACCACATGGAAACCTCCCTGAAGGATTTTGAAACTATCCTTCACTTAATTAATAAGGAG<br>GAGATTGACTCAAATGACACCATTCATATGACCGCCAACGAAAATATTATGTCTAAATTGTCCAAACACTACT<br>TAAAAAGCACTTTGTCTTACCGCTACCATGTCGGAATGTTCGATGATCAAAAGAACCTGACAGTCTCGCGTT<br>CGTGTCTTATCAAAAACTCTTTGATGCTGCGTTGCCTTTCACCCATCTTCCTGTTAGAACAACAAGCCCGTG<br>AATACGTAAAAAAAATGTTCTTCGCTGAGTATGCGGACTTTCGTCCTTTGTCCGGTATGCACACCGTTTTTTG<br>TATCTTATCTACCTTAACAAAACCGAACGATCGTGTCTATGTCTTCACGACCGAATCGGTAGGACACGCAGC<br>CACAGTTTCTTTATTGAAGTCGTTGGGTCGCAAAGTGTCCTTCATCCCATTTTGTGAGAAGAAACTTGATATT<br>GACTTAGAGAAGCTGAGTAAACAAATCTTGATTGAGAAACCCAACGCAATTCTTTTTGATTTTGGTACTCCAT<br>TCTACCCATTGCCGATCCGCGAAATTCGCGAGATTGTAGGAAACGACGTGAAGATGATTTATGACGCCTCG<br>CATGTGTTGGGTTTGATTGCGGGTGGACAGTTCCAAAATCCACTTCTTGAAGGCTGTGACGTGCTGATCGG<br>AAATACTCACAAGACATTTCCGGGGCCGCAGAAAGGCATGATCTTGTATAAAAACAAGTCTTTGGGAAAGGA<br>GATCGCAACAGAAATTTTCAAATCAGCCATTTCTGCGCAGCATACTCATCATGCTATCGCCCTGTACGTTAC<br>TATCATTGAAATGTATATCCACGGGAAGGAATACGCCAACCAAATCATCAAAAATAATCATGCGTTATCCCAG<br>GCATTAATCAATGAAGGTTTTAAAATTTTTAAGCGTAAAAACCAGTTTAGCCTTAGTCACATGATTGCGATTA<br>CGGGGGATTTTCCGATTGATCATCATGTTGCATGTGCCGATTTGCATAATTCTAACATCTCCACAAATTCGC<br>GTATTCTGTATGACTTTCCAGCCGTGCGCATTGGCGTTCAGGAGGTTACACGTAAAGGAATGAAAGAAAAG<br>GATATGGTGCAATTAGCCAAATTTTTTAAGGAAATCATCCTGGATCGCAAGAACATCAGCTCTAAAATCAAG<br>GAGTTCAATAACAAATTCAATAGTATTGAATATAGTCTTGACGAGATCTACGAGAAGTTATTCTAACTTGATG<br>GGGGATCCCATG
**DbTTA**	MBI5609283	CCATCACCATCATCACCACTTGACGAATAATCGCGAGCTTATGGACCGTATCGGTTATAATCTTTCACAAGG<br>TTTAGTTTCAAGCCAGCATACCGCAAGTCTGGTCGCTTTATTTATTGCATTACATGAAGCACGCCTGACCGG<br>CAAAGCGTTCGCAAAGCAAGTGGTAGAAAACGCCCGTACGTTGGCGAGTCGTTTGGCGGCACTTGGCGTT<br>CCGGTGTTAGCGCGTTCAGATGGCCAGTTTACCGACAATCATCATTTCTTCATCAATTTGACCGGCGTGGC

		GAGTGCTCCTCACCAAATGGAGCGCTTACTTCGTGCCCATTTGGTTGTTCAGCGCGGCATGCCGTTTCGCA<br>ACGTTGACGCCTTGCGTGTTGGCGTGCAAGAAGTCACACGCCGCGGTTATGGACCCGGCGAGATGGCGCA<br>GCTGGCAGAGTGGATTGCGTCAATCGTCATCGGCGGTGCGGACCCCGAGGTAGTAGCACCTGCCGTGCAA<br>GCCATGGCTAAGCGCTTTGACACTATCTATTATACGGGCGAAACGGTGGACGGTAAACTTGATCTTCCAGA<br>AATCGCAGCGCCGAGCGCTAAGGGCCGTTGGGTTGACTATCGCCATTTGGGAAATGATTTTGCAATGGACG<br>ATACTGAGTTCTCCGAAATTCGCGCCTTGGGTGCTGCCGCGGGAGCCTTCCCAAACCAGACCGACAGTACA<br>GGTAACGTCTCGTTACGTTCAGGAGCCCGTGTATTCGTGTCGTCTAGCGGGTCATATATTAAGCACCTGGC<br>CGACGGACAGGTCGTCGAGTTGGACGCGGTAGATCCCTCAGGGGAATTGATTGACTATCATGGTGCGGCG<br>TTGCCCAGCAGTGAGAGTCTGATGCACTTCTTAGTTTACCAGAATGTGCCAGCGGGCGCAGTTGTGCACAC<br>TCACTATTTATTAACCAACCAAGAGGCTGCCGACTTCGATGTGGCGGTGATCGCTCCTCAGGAATATGCCA<br>GTATTGCACTTGCCCGCGCAGTAGCAGAAGCCAGTAAACGCTCCCGTATCGTGTATATTCAAAAACACGGA<br>TTAGTGTTTTGGGGTACAGACACTGCAGATTGTCTGTCTCAGGTTCACAACTTTATTCACAACCGTCCAAATC<br>GTCGCGCAGCTGAGGCGGTCTATGCCTCTTAACTTGATGGGGGATCCCATG
**SUMO-tag**		<u>ATG</u>TCCCTGCAGGACTCGGAGGTTAACCAGGAAGCAAAGCCGGAAGTCAAACCGGAAGTGAAACCCGAAA<br>CTCACATCAATCTGAAGGTAAGTGATGGTTCTTCAGAGATATTCTTTAAAATTAAAAAAACCACGCCTCTGCG<br>GCGTCTTATGGAAGCGTTCGCCAAACGACAAGGGAAAGAGATGGATAGCTTACGTTTTCTCTATGATGGCA<br>TTCGCATCCAGGCGGATCAAGCTCCAGAGGACTTGGATATGGAAGATAACGACATTATCGAAGCCCATCGC<br>GAACAGATTGGTGGC

*For StTTA, we arbitrarily cloned a variant to contain a 36-residue truncation from the N-terminus (StTTA-Δ36) such that its new N-terminal residue would align with the sequence of ObiH and the other candidate TTAs.

III. Supplementary Figures

Figure S1

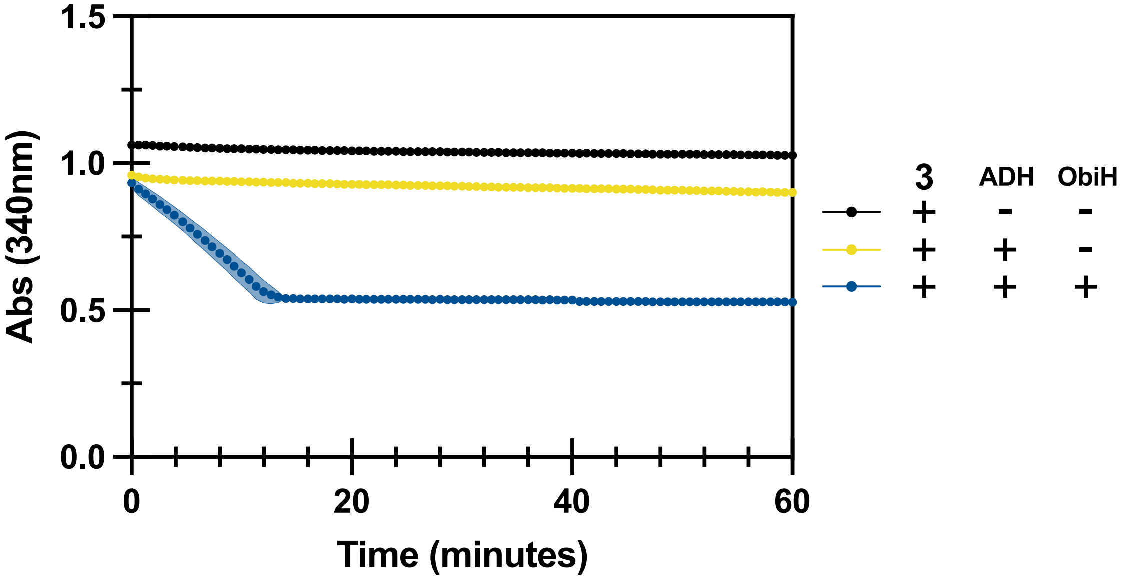

Figure S1: TTA-ADH coupled assay optimal output. The observed depletion of absorbance at 340nm for the condition TTA, ADH, and aldehyde compared to no depletion for negative controls with no enzyme, and no TTA. Assay performed in triplicate with shading representing the standard deviation.

Figure S2

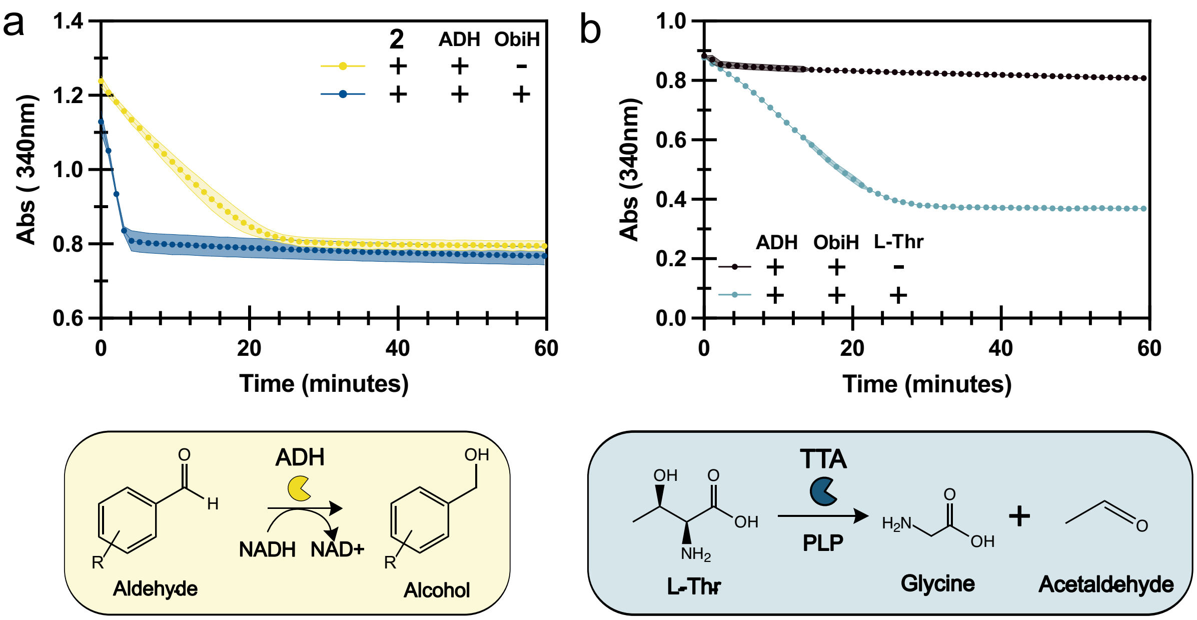

Figure S2: TTA-ADH coupled assay background activity and reactions. (a) Background activity of the ScADH on an aromatic aldehyde of interest leading to a depletion of NADH and corresponding loss of absorbance. Assay performed as described in the main text using 1 mM aldehyde and 1.45 μM ObiH. (b) Background activity observed from the L-threonine reacting with the TTA and releasing acetaldehyde that is then consumed by the ADH. Assay performed as described in the main text using 12.4 μM ObiH. Assays performed in triplicate with shading representing the standard deviation.

Figure S3

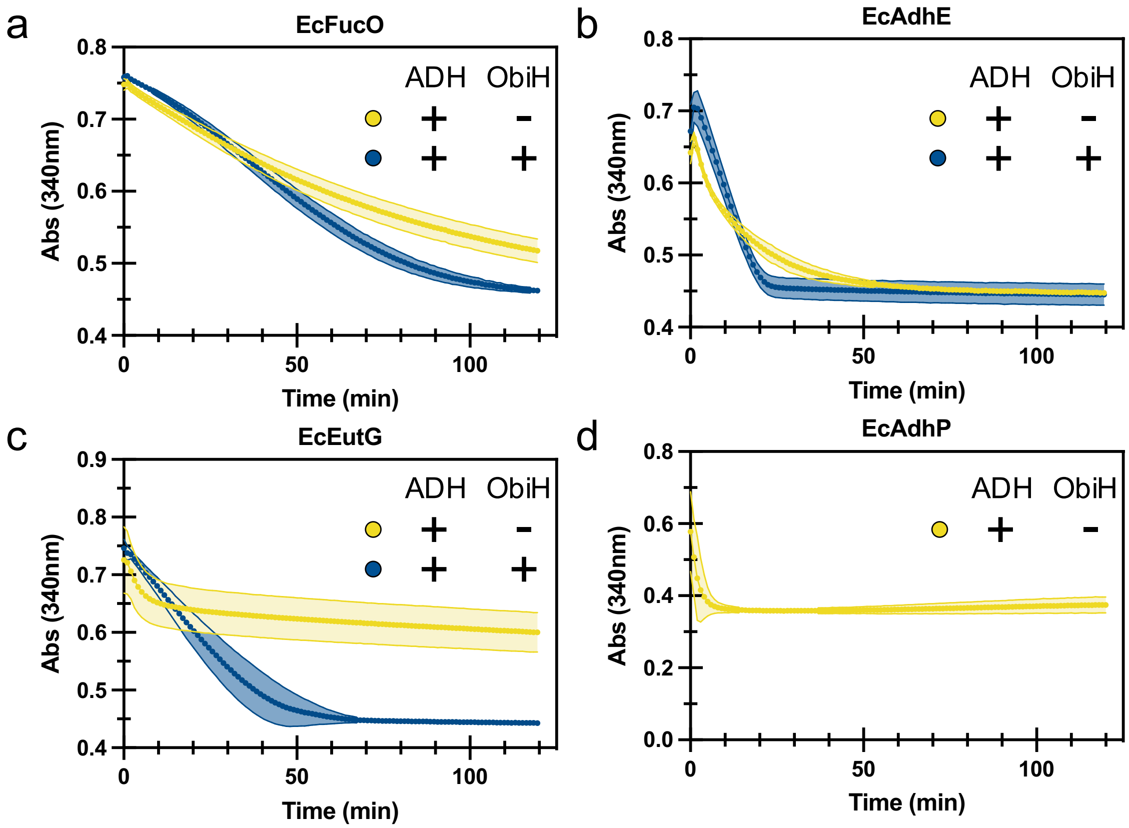

Figure S3: Assessing alternate ADHs for background aldehyde activity. In vitro TTA-ADH coupled assay with 4 ADHs from *E. coli* using **1** as a substrate. Each has confounding activity with ADH only control, so they were not pursued further. The final ADH concentration in the assay is listed in parenthesis following the protein name. (a)EcFucO (230 µg/mL) (b) EcAdhE (200 µg/mL) (c) EcEutG (100 µg/mL) (d) EcAdhP (100 µg/mL) which had such a rapid rate that we did not screen it in the presence of ObiH. Assay performed in triplicate with shading representing the standard deviation.

Figure S4

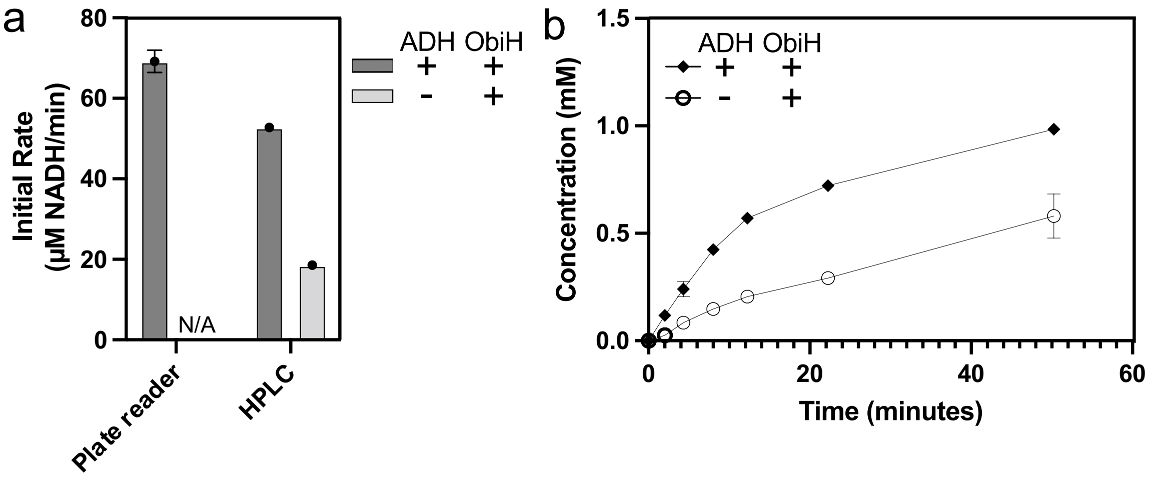

Figure S4: Validation of the TTA-ADH assay using HPLC analysis. (a) A comparison of the rates measured using the continuous plate reader assay and a discontinuous HPLC assay to validate the rates observed on the plate reader are accurate. **3** was used as the substrate with all other conditions described in the methods of the main text and SI. In addition to the time course assay with ObiH and ADH, the assay was also performed with only ObiH to understand the impact of the ADH on the reaction rate. Plate reader rates performed and calculated in triplicate with error bars representing the standard deviation. Discontinuous HPLC assay performed in triplicate with rate calculated using the averages for each triplicate. (b) The concentration of β-OH nsAA as a function of time for the HPLC-based time course assay. Assay performed in triplicate with error bars representing standard deviation.

Figure S5

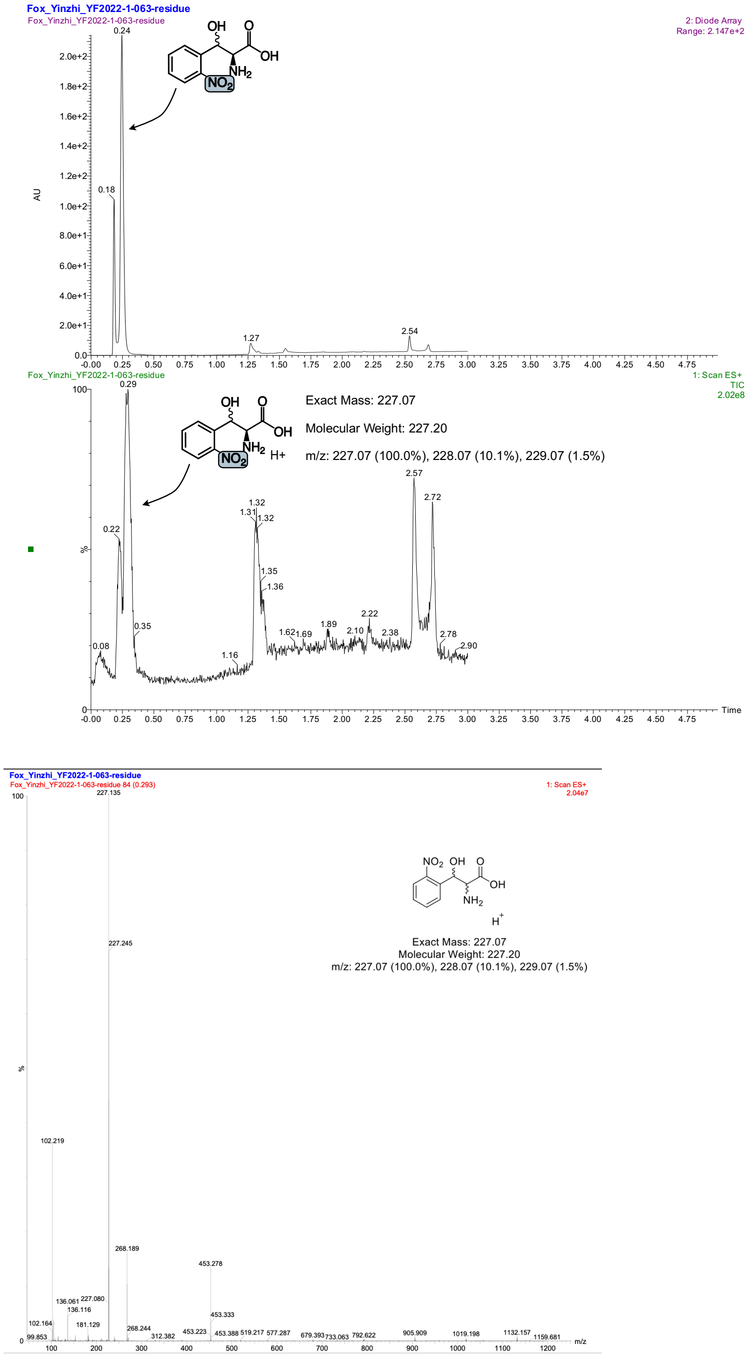

Figure S5: LC-MS for synthesized 2-nitro-*β*-OH-phenylalanine. The top two charts represent the raw spectra, and the bottom chart is the specific mass for the peak highlighted. The bottom chart shows mass spectra (m/z) extracted at the highlighted elution time.

Figure S6

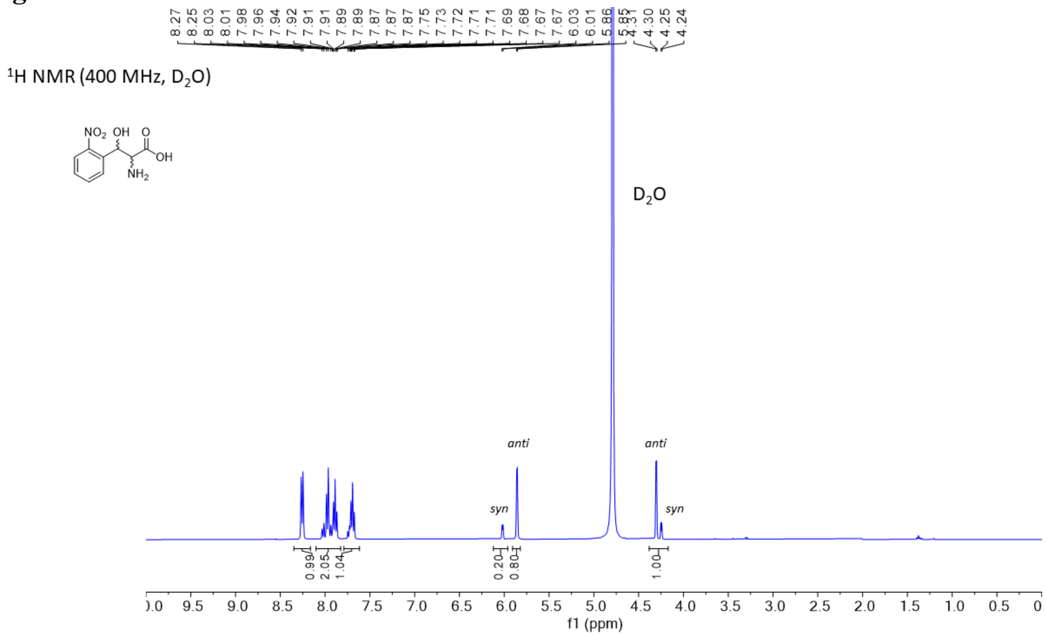

Figure S6: ^{1}HNMR for chemically synthesized 2-nitro-*β*-OH-phenylalanine. NMR spectra to confirm the chemical synthesis of 2-nitro-*β*-OH-phenylalanine.

Figure S7

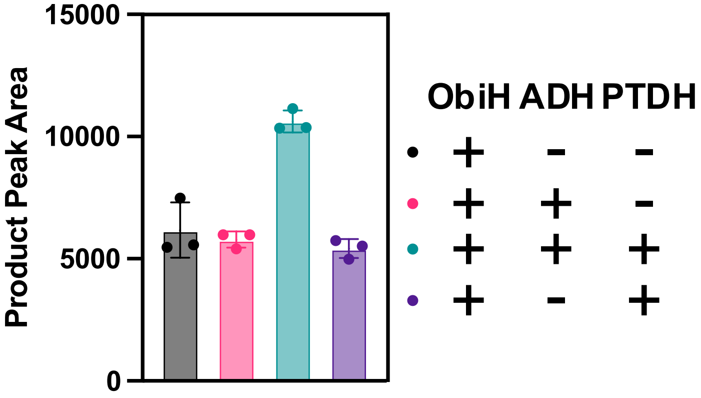

Figure S7: Coupling ADH with co-factor regeneration for improved β-OH-nsAA yields. As previously shown[5], coupling the TTA to an ADH and a recycling system will improve product yields. We observed higher conversion when coupling ObiH, ADH, and an engineered phosphite dehydrogenase (PTDH)[6] and 10 mM of **3.** Product concentrations were measured after 24 h using the reaction conditions described in the SI. Peak area is calculated as the area under the curve for the absorbance spectra output from HPLC. Experiment performed in triplicate with each replicate represented and error bars represent the standard deviation.

Figure S8

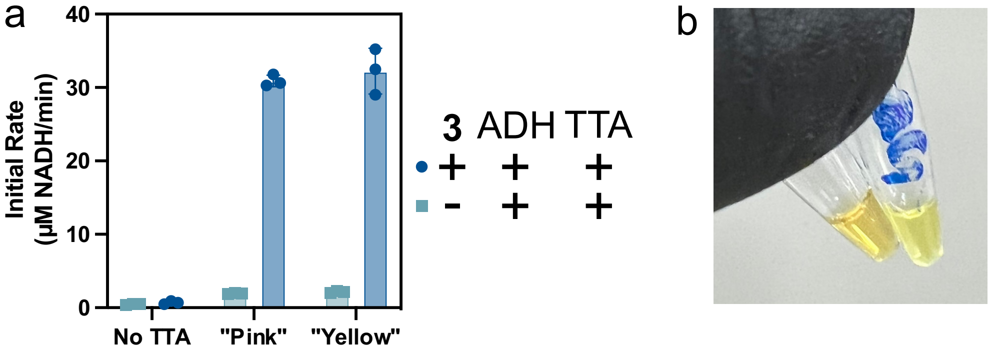

Figure S8: Screening photo-treated TTA in the TTA-ADH coupled assay. (a) The literature suggests that ObiH is in its most active form after being activated with light[7]. We used the TTA-ADH coupled assay to determine if there were any differences with photo-treated enzymes by using a sample directly from the dark -80 °C storage, "Pink", and another, that had been sitting on ice under ambient light, "Yellow", for 4 h. The rates measured with the TTA-ADH coupled assay were very similar between the two enzymes samples. Assay performed in triplicate with each replicate represented and the error bars represent the standard deviation. (b) The light exposure did change the color of the sample which we used as verification of photo-treatment prior to beginning the coupled assay. Since we did not observe differences in behavior, we proceeded with our assays without photo-treatment. We hypothesize that this is because of the extensive light exposure that occurs during the purification process.

Figure S9

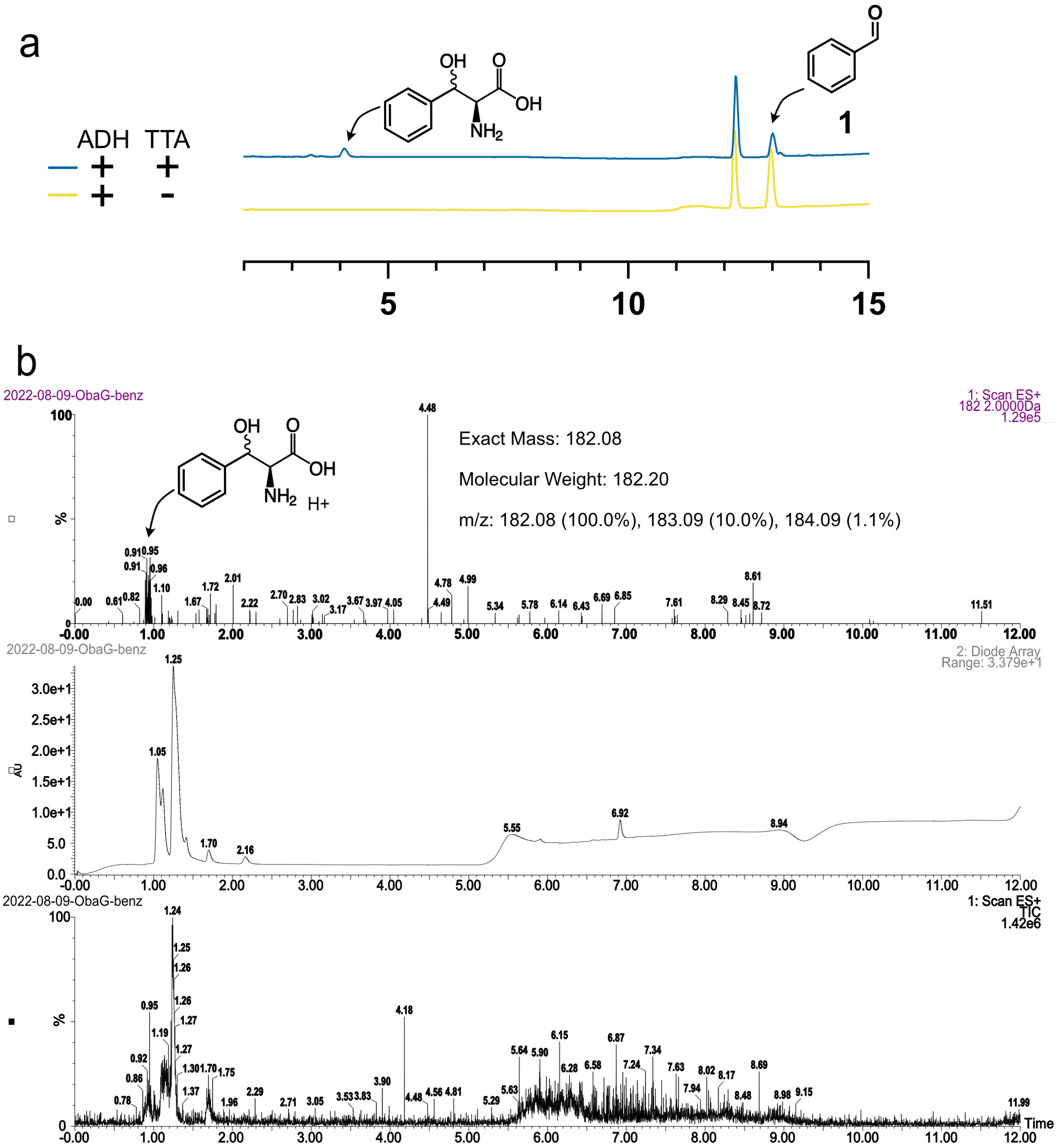

Figure S9: HPLC and LC-MS confirmation for β-OH-nsAA produced from benzaldehyde (1). (a) HPLC traces at 210 nm for the with and without TTA conditions. (b) LC-MS trace.

Figure S10

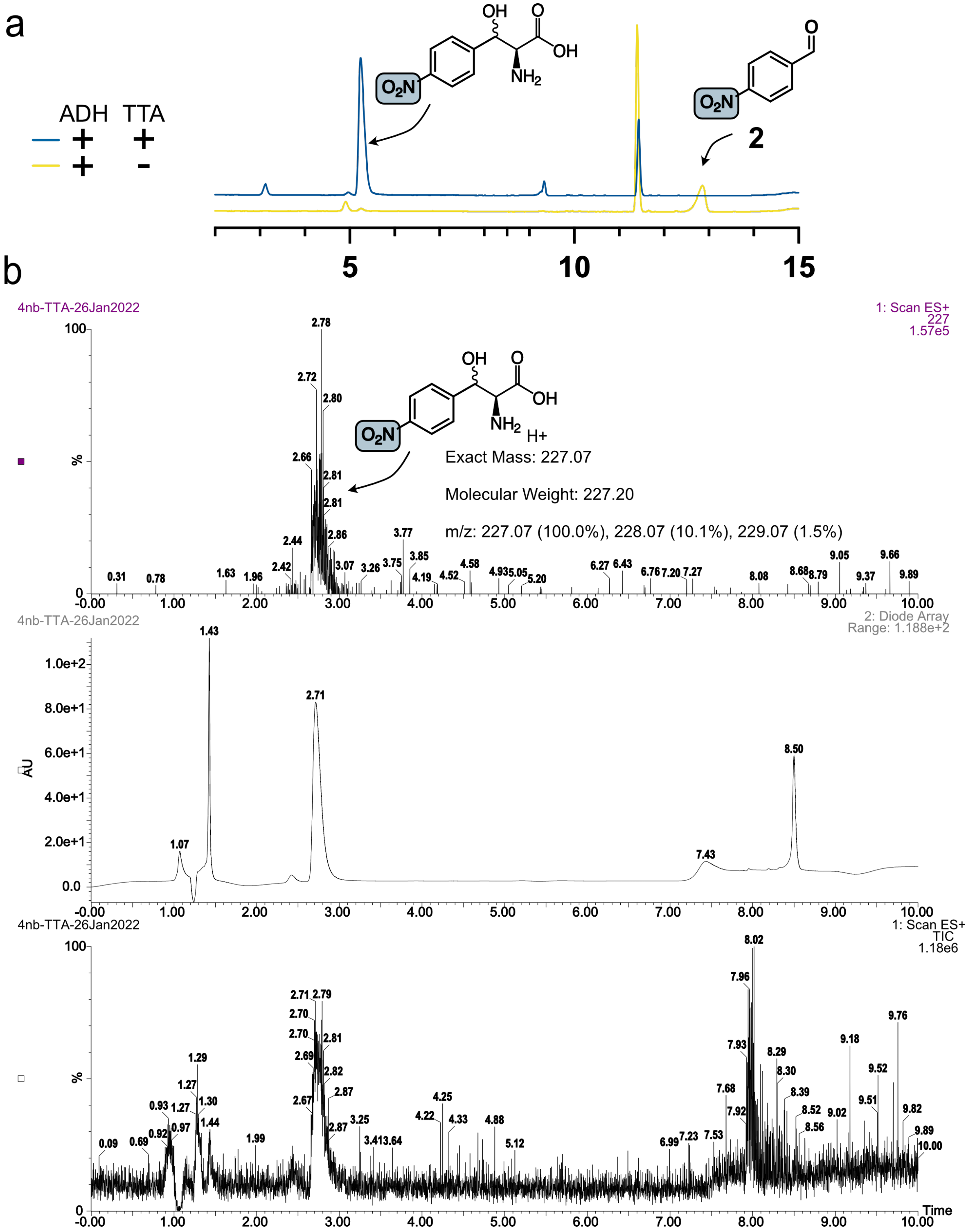

Figure S10: HPLC and LC-MS confirmation for β-OH-nsAA produced from 4-nitro-benzaldehyde (2). (a) HPLC traces at 280 nm for the with and without TTA conditions. (b) LC-MS trace.

Figure S11

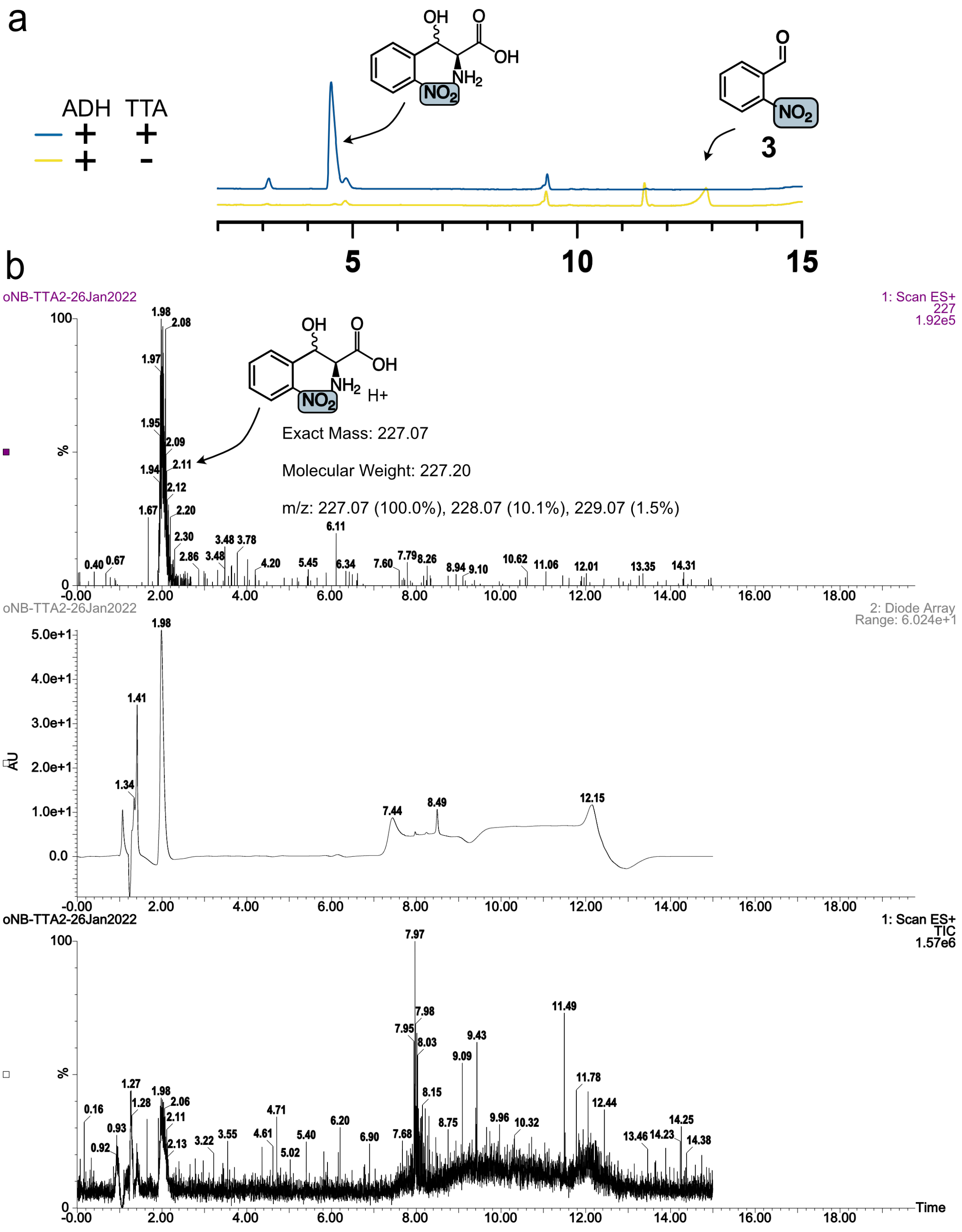

Figure S11: HPLC and LC-MS confirmation for β-OH-nsAA produced from 2-nitro-benzaldehyde (3). (a) HPLC traces at 280 nm for the with and without TTA conditions. (b) LC-MS trace.

Figure S12

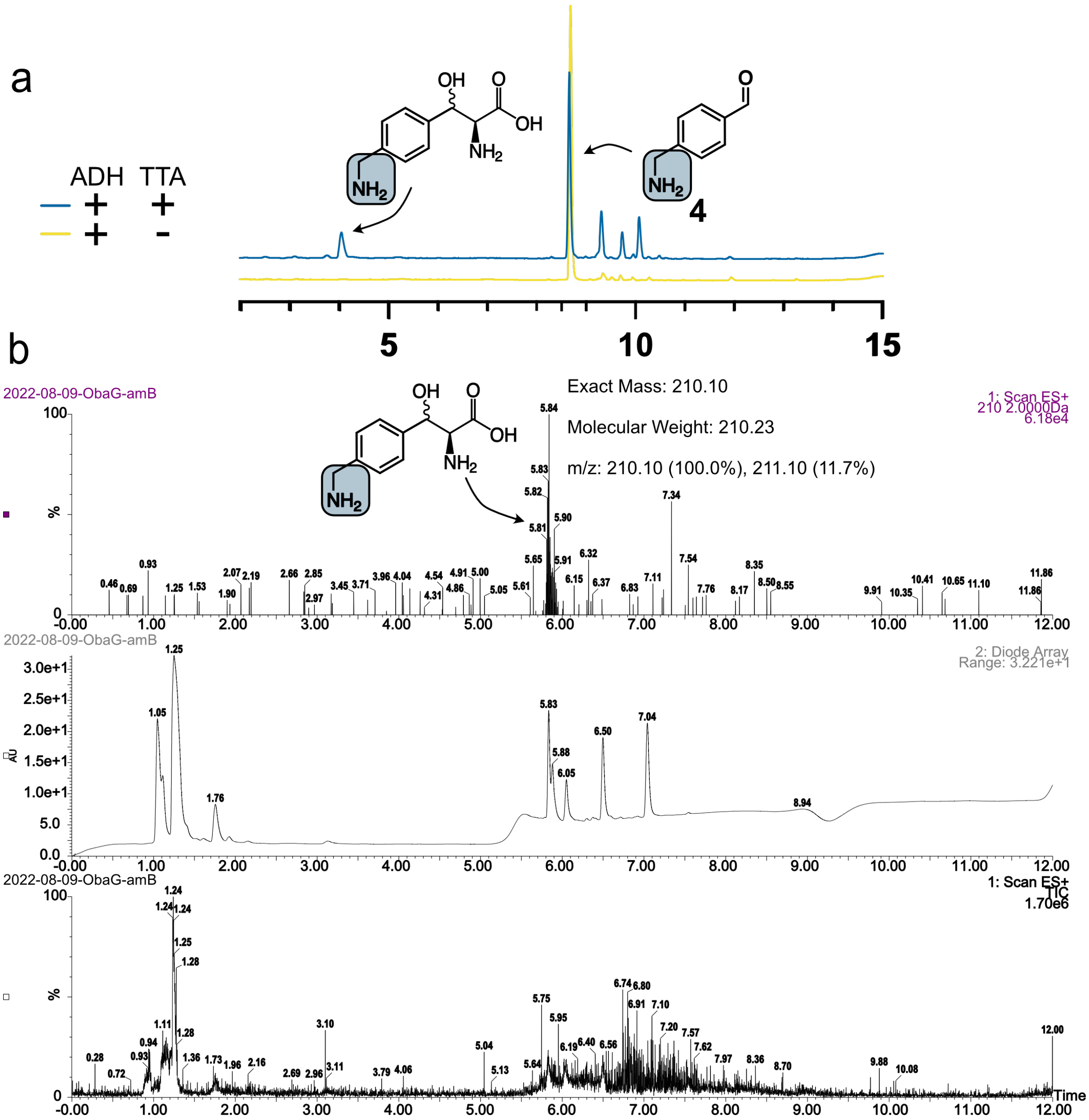

Figure S12: HPLC and LC-MS confirmation for β-OH-nsAA produced from 4-amino-methyl-benzaldehyde (4). (a) HPLC traces at 280 nm for the with and without TTA conditions. (b) LC-MS trace.

Figure S13

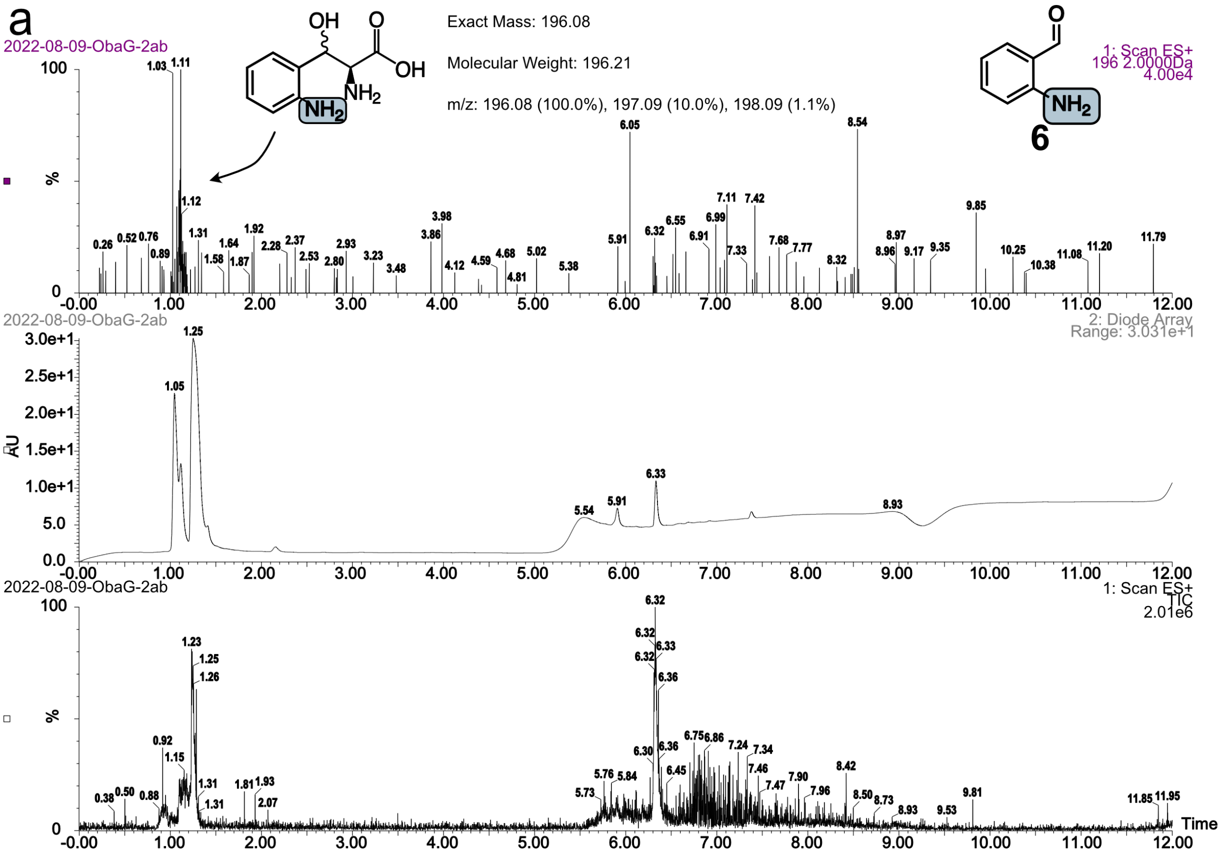

Figure S13: LC-MS confirmation for β-OH-nsAA produced from 2-amino-benzaldehyde (6). (a) LC-MS trace. It was difficult to detect 2-amino-benzaldehyde via HPLC due to its co-elution with the solvent front.

Figure S14

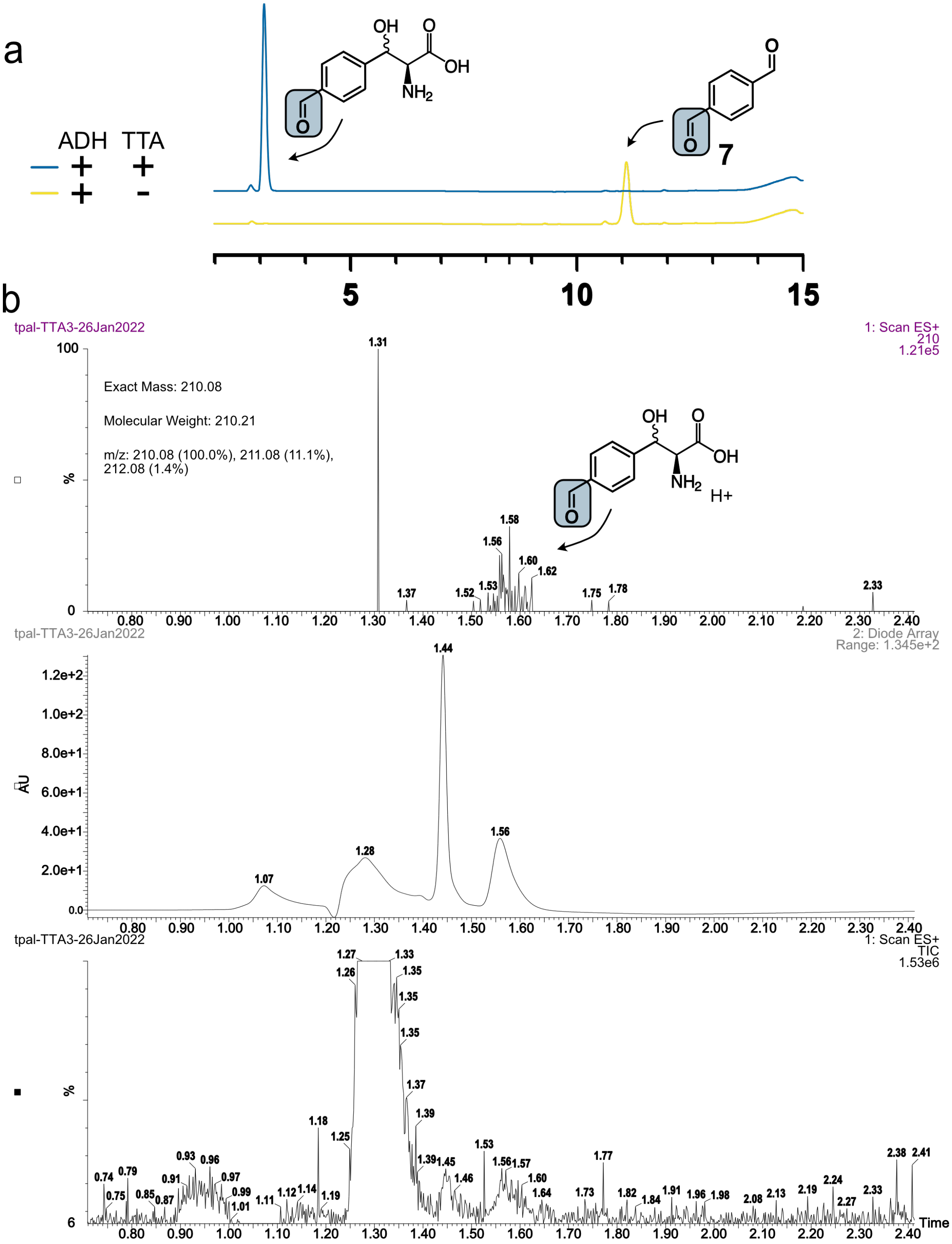

Figure S14: HPLC and LC-MS confirmation for β-OH-nsAA produced from terephthalaldehyde (7). (a) HPLC traces at 250 nm for the with and without TTA conditions. (b) LC-MS trace.

Figure S15

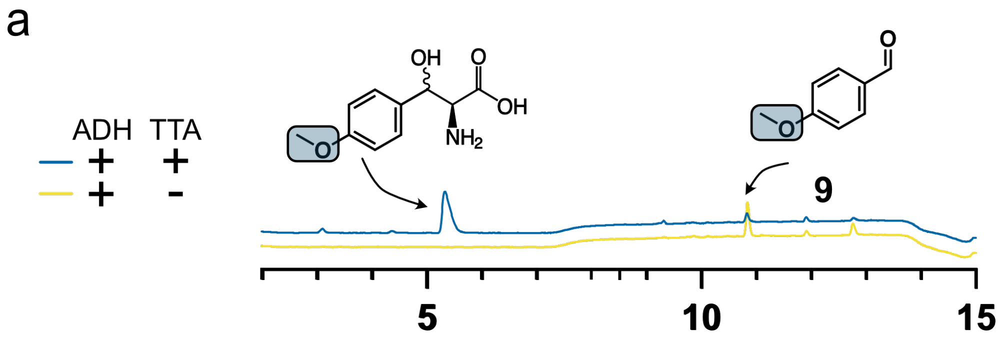

Figure S15: HPLC confirmation for β-OH-nsAA produced from 4-methoxybenzaldehyde (9). (a) HPLC traces at 210 nm for the with and without TTA conditions. Despite the robust peak formed on HPLC, we had difficulty verifying the product via LC-MS, possibly because it does not ionize well.

Figure S16

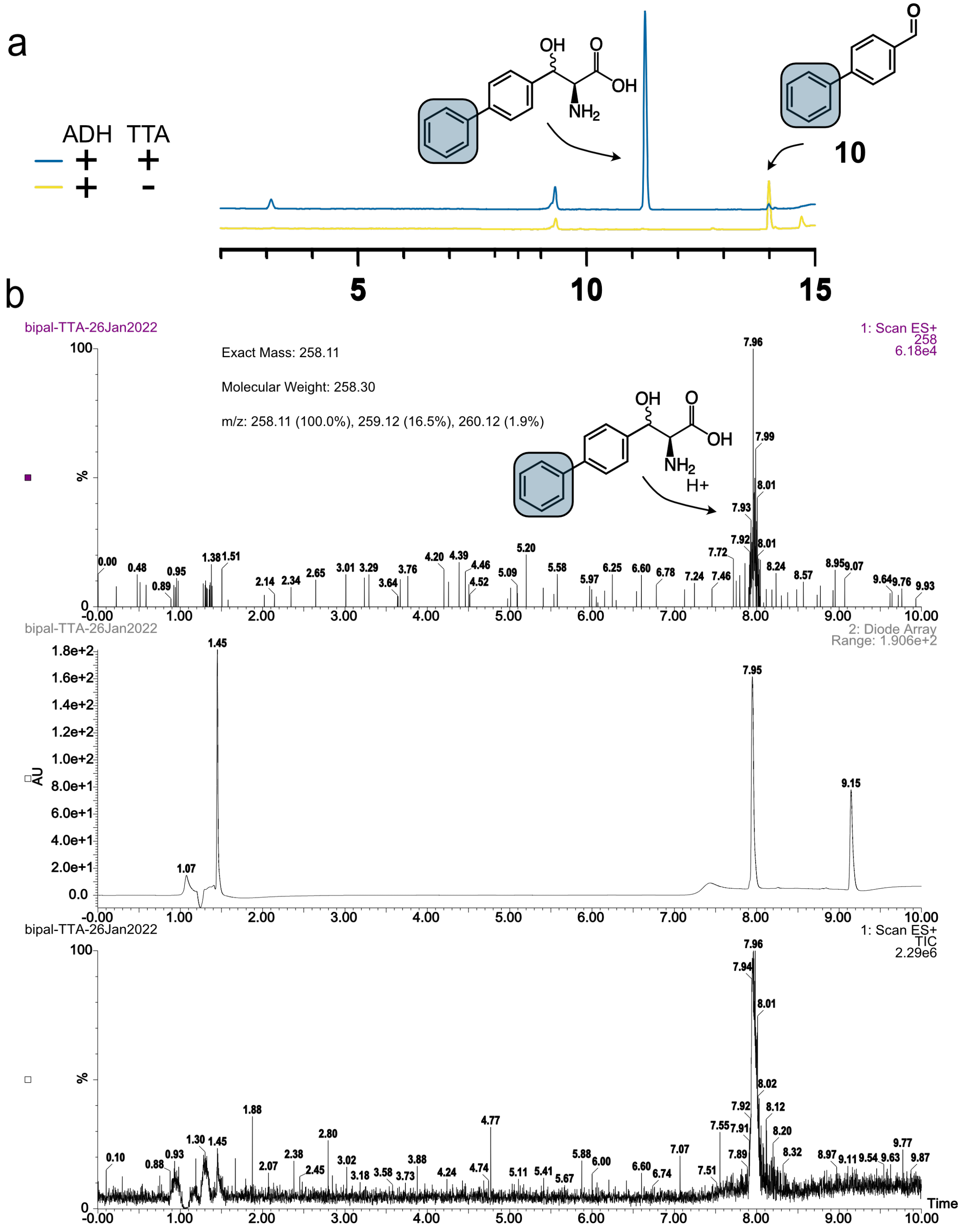

Figure S16: HPLC and LC-MS β-OH-nsAA produced from confirmation for 4-biphenylcarboxaldehyde (10). (a) HPLC traces at 280 nm for the with and without TTA conditions. (b) LC-MS trace.

Figure S17

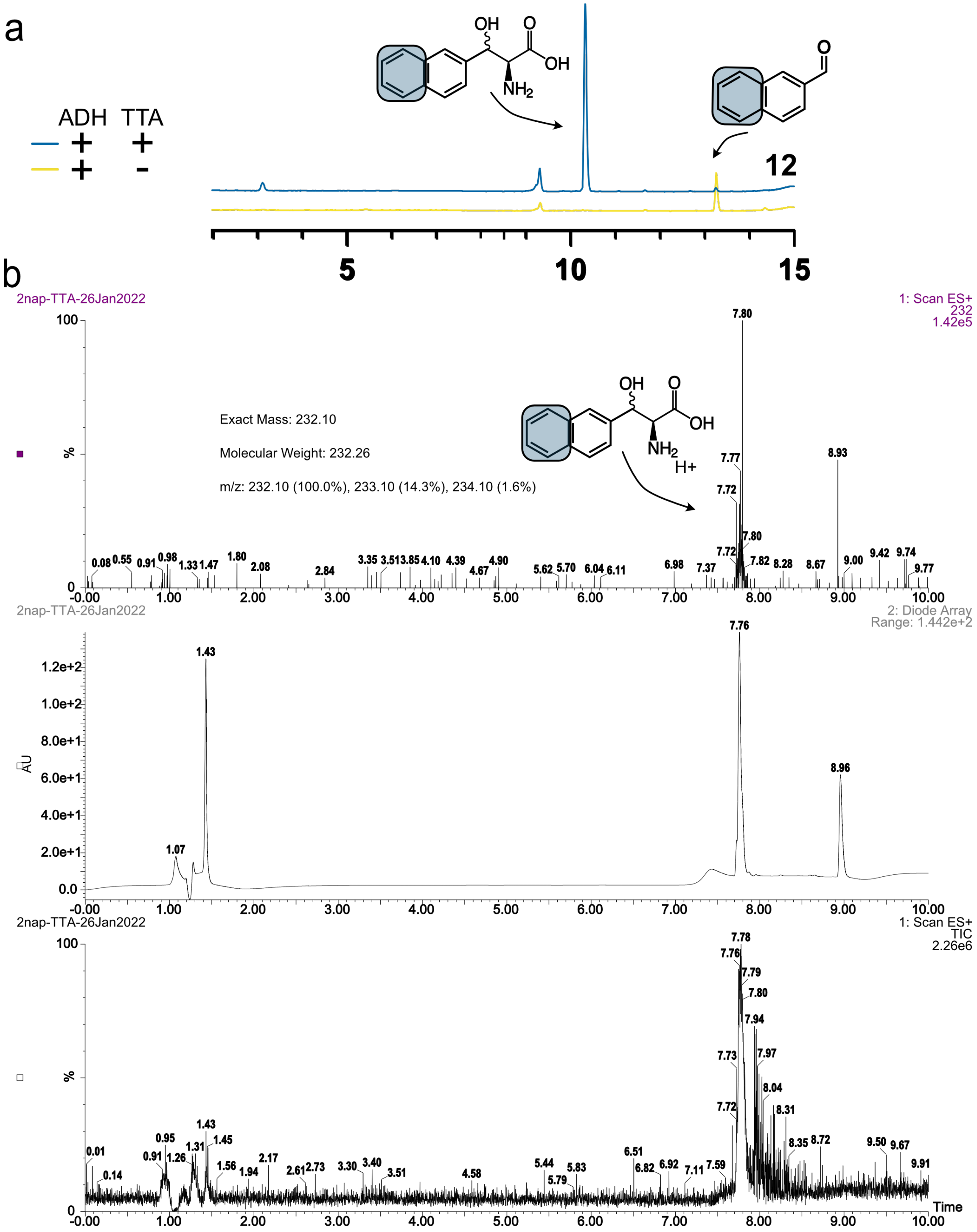

Figure S17: HPLC and LC-MS confirmation for β-OH-nsAA produced from 2-napthaldehyde (11). (a) HPLC traces at 280nm for the with and without TTA conditions. (b) LC-MS trace.

Figure S18

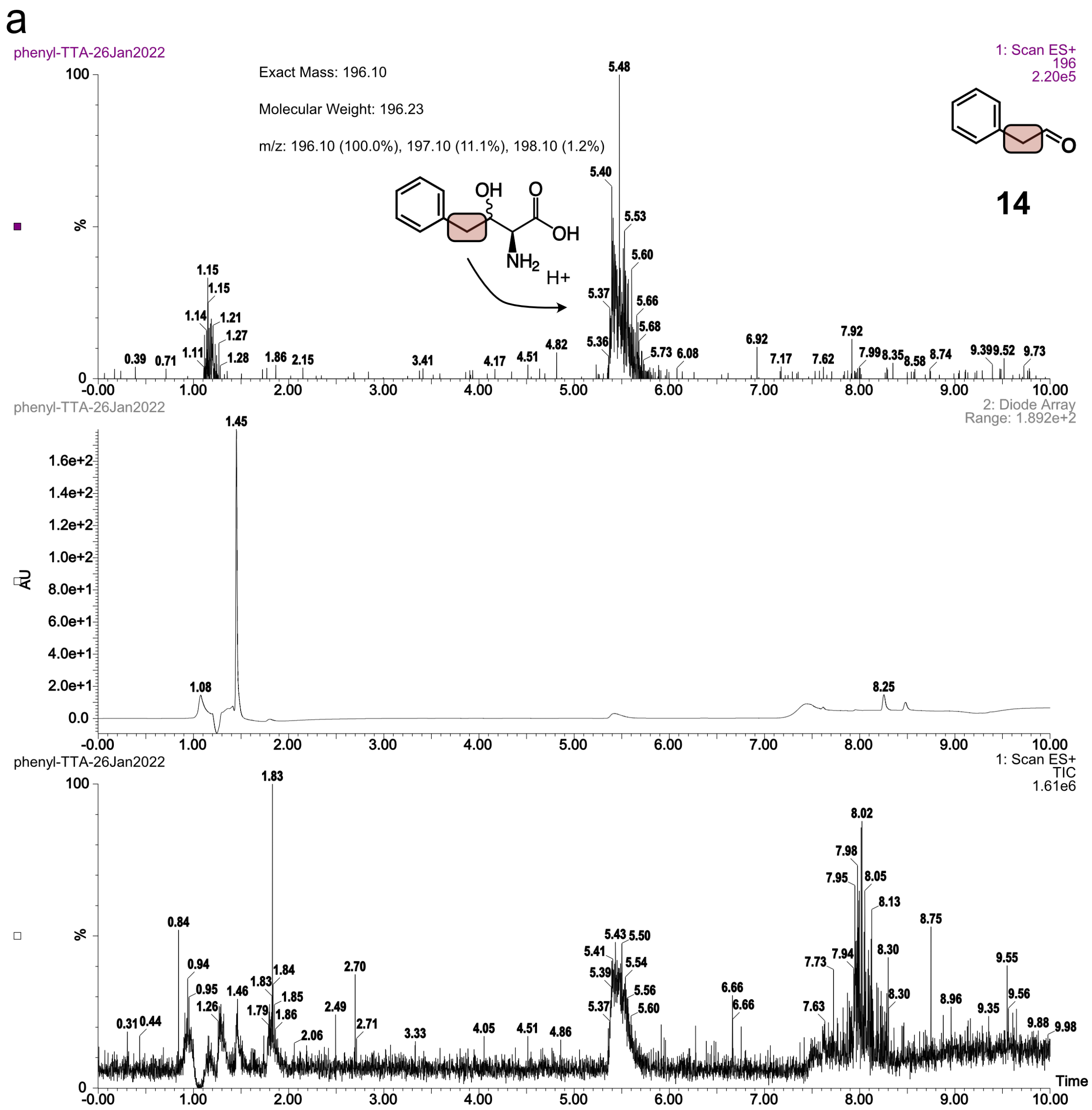

Figure S18: LC-MS confirmation for β-OH-nsAA produced from phenylacetaldehyde (14). (a) LC-MS trace. We were unable to detect the product via HPLC due to low absorbance across all wavelengths screened.

Figure S19

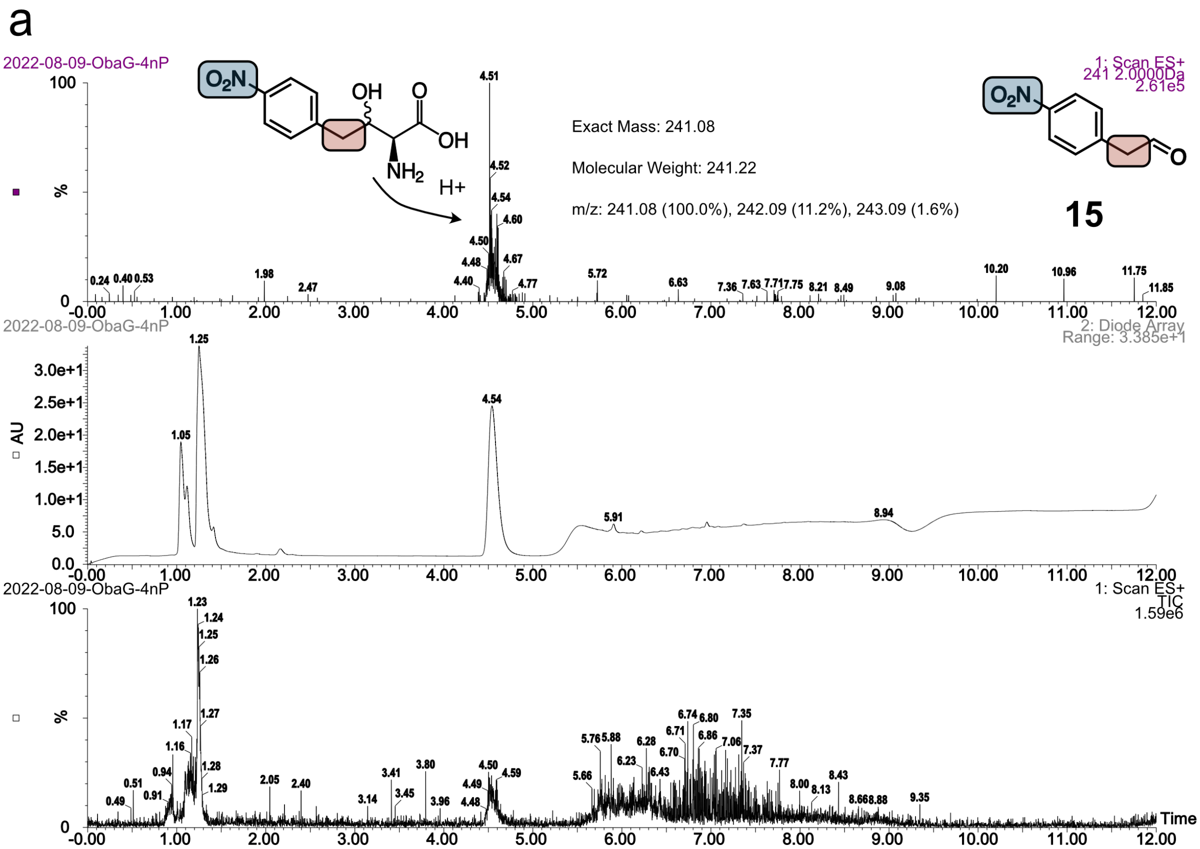

Figure S19: LC-MS confirmation for β-OH-nsAA produced from 4-nitro-phenylacetaldehyde (15). (a) LC-MS trace. It was difficult to observe a specific product peak on HPLC because of the instability of 4-nitro-phenylacetaldehyde.

Figure S20

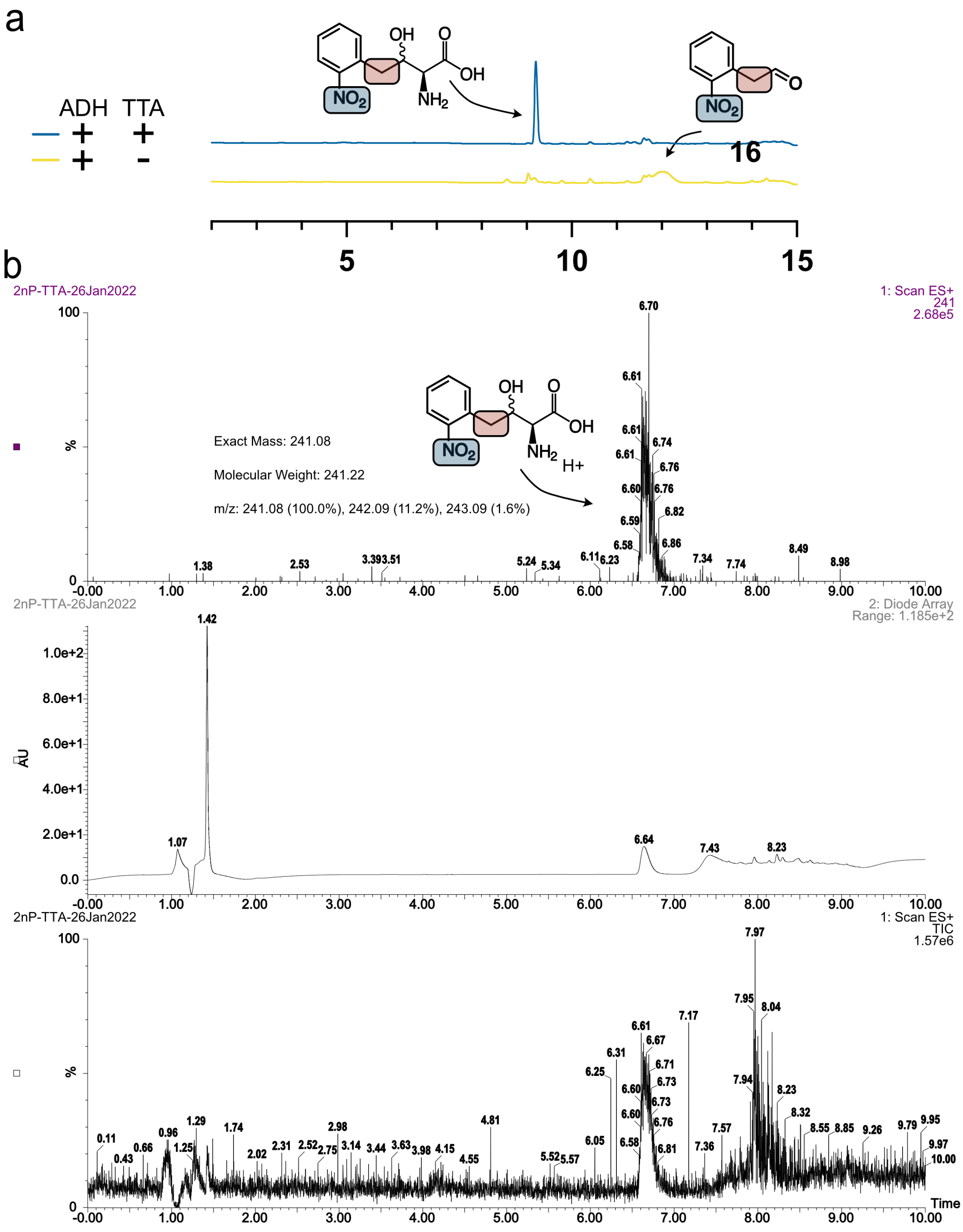

Figure S20: HPLC and LC-MS confirmation for β-OH-nsAA produced from 2-nitrophenylacetaldehyde (16). (a) HPLC traces at 280nm for the with and without TTA conditions. (b) LC-MS trace.

Figure S21

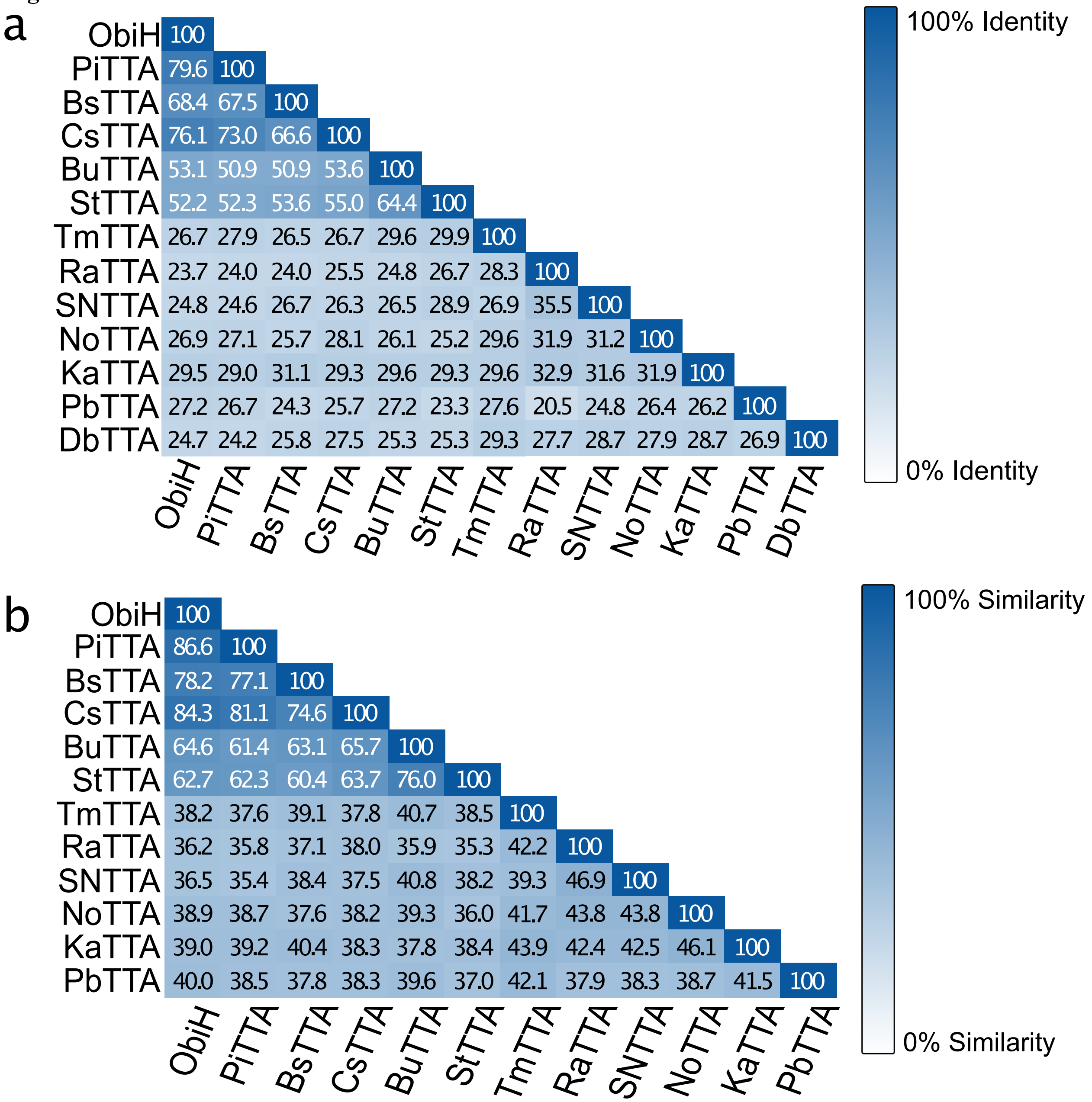

Figure S21: Sequence identity and similarity matrix. (a) Identity matrix from Figure 3B with % values. (b) Similarity matrix for all TTAs except DbTTA due to poor alignment[8]. Similar residues are considered the following: GAVLI, FYW, CM, ST, KRH, DENQ, P.

Figure S22

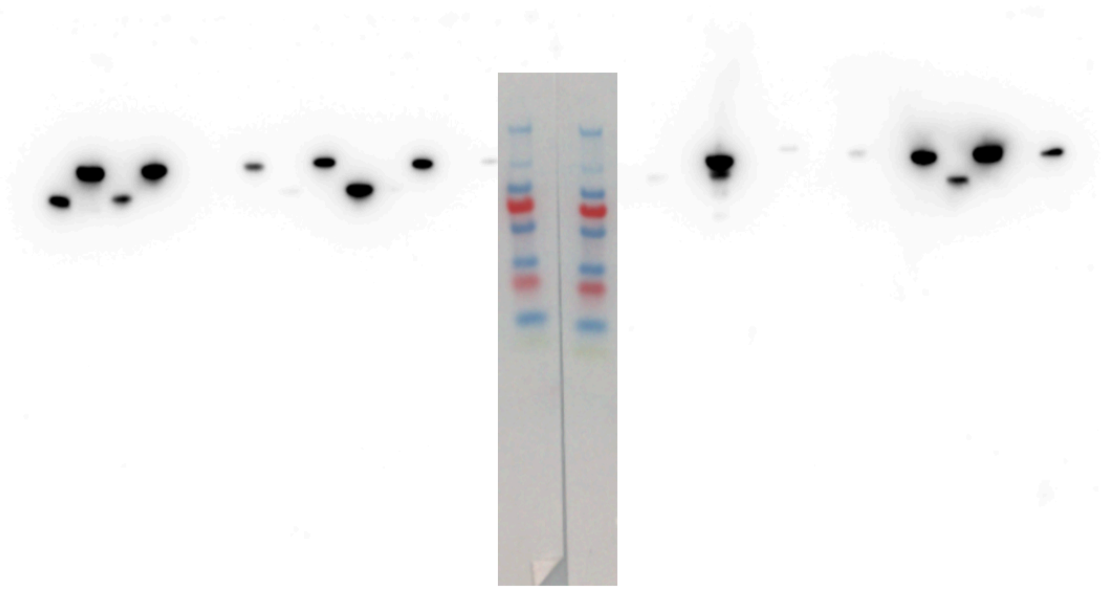

Figure S22: Unedited western blot from Figure 3b. Unedited image of the western blot in Figure 3b comparing the expression of TTAs with and without a SUMO-tag. The protein ladder is the Thermo Scientific™ Spectra™ Multicolor Broad Range Protein Ladder.

Figure S23

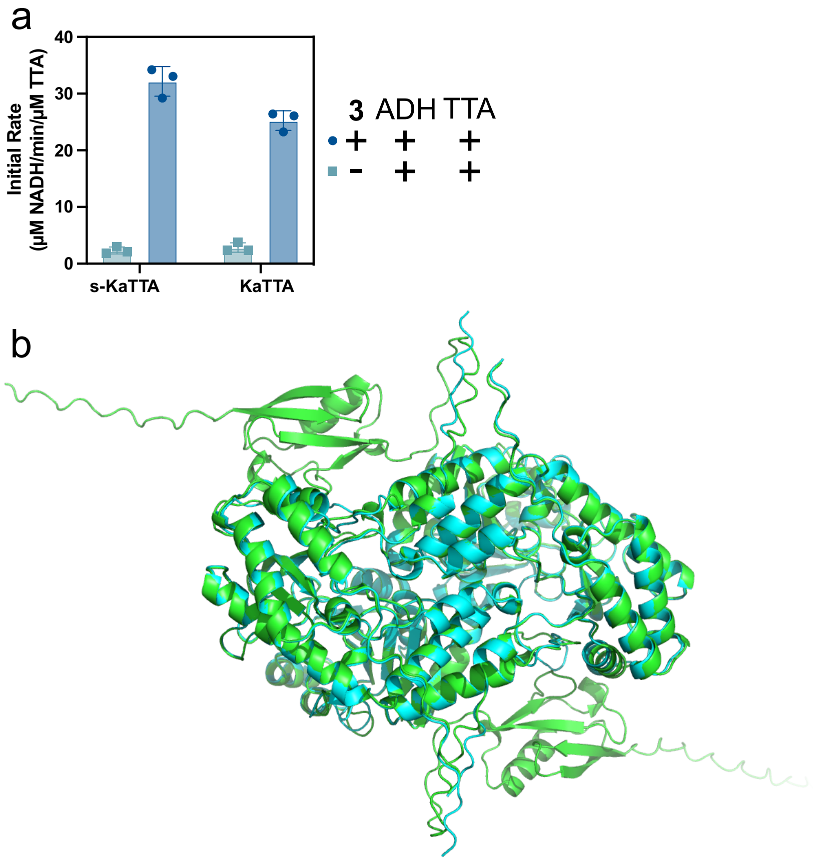

Figure S23: SUMO versus no SUMO tag for KaTTA. (a) Rates from the TTA-ADH coupled assay for KaTTA with and without the SUMO tag. (b) Structural overlay of KaTTA with (green) and without (blue) the SUMO-tag. Structures were generated using AlphaFold2 Colab[9] notebook and aligned using PyMOL.

Figure S24

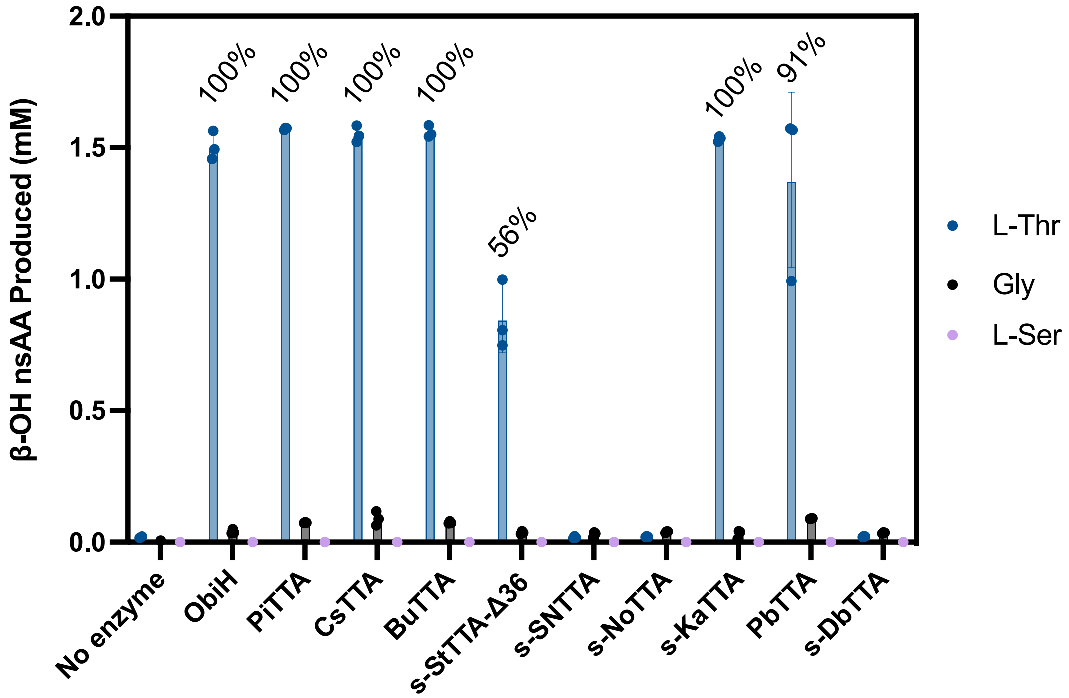

Figure S24: L-Thr is required for β-OH-nsAA production with the active TTAs. Conversion of **3** to the β-OH-nsAA only occurs in the presence of L-Thr for the active TTAs. We observed product formation for StTTA-Δ36 indicating it is also active with significant β-OH-nsAA formation after 20 h incubation. Reaction conditions described in the SI Methods. While there appears to be some small product formation in the presence of glycine, these peaks appeared in the negative control without enzyme. We hypothesize they are from the acid-catalyzed imine formation from the unreacted aldehyde and glycine. We did not observe imine formation in the negative controls containing L-Thr and L-Ser. Assay performed in triplicate for L-Thr and Gly with all replicates represented and error bars that represent the standard deviation. Assay for L-Ser was not assayed in triplicate because there was no peak formation. The percentage above the L-Thr bars indicates the analytical percent conversion observed for that reaction.

Figure S25

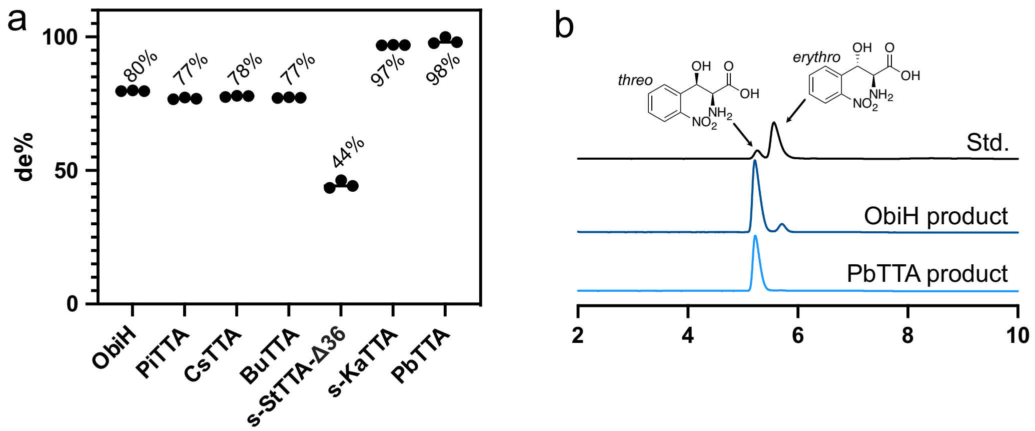

Figure S25: de% for β-OH-nsAA produced from 2-nitro-benzaldehyde for all active enzymes. (a) The de% for the *threo* isomer for each of the active enzymes with reaction conditions as specified in the main text and quenched after 20 h. de% was calculated as follows (*threo* - *erythro*)/(*threo* + *erythro*). (b) HPLC traces for ObiH and PbTTA as well as the chemically synthesized standard to demonstrate how we identified the diastereomers.

Figure S26

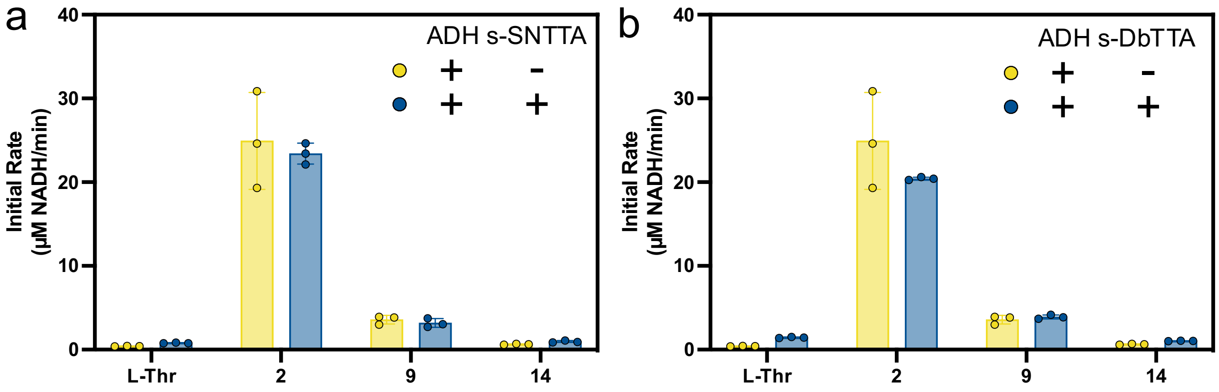

Figure S26: Negative results for activity of SNTTA, and DbTTA with additional aldehyde substrates. Initial rates for a series of different aldehyde substrates using L-Thr with (a) SNTTA and (b) DbTTA. The rates are approximately equal in the "no TTA" and "TTA + ADH" cases for all aldehyde substrates tested, indicating there is no activity of SNTTA or DbTTA on the aldehyde substrate.

Figure S27

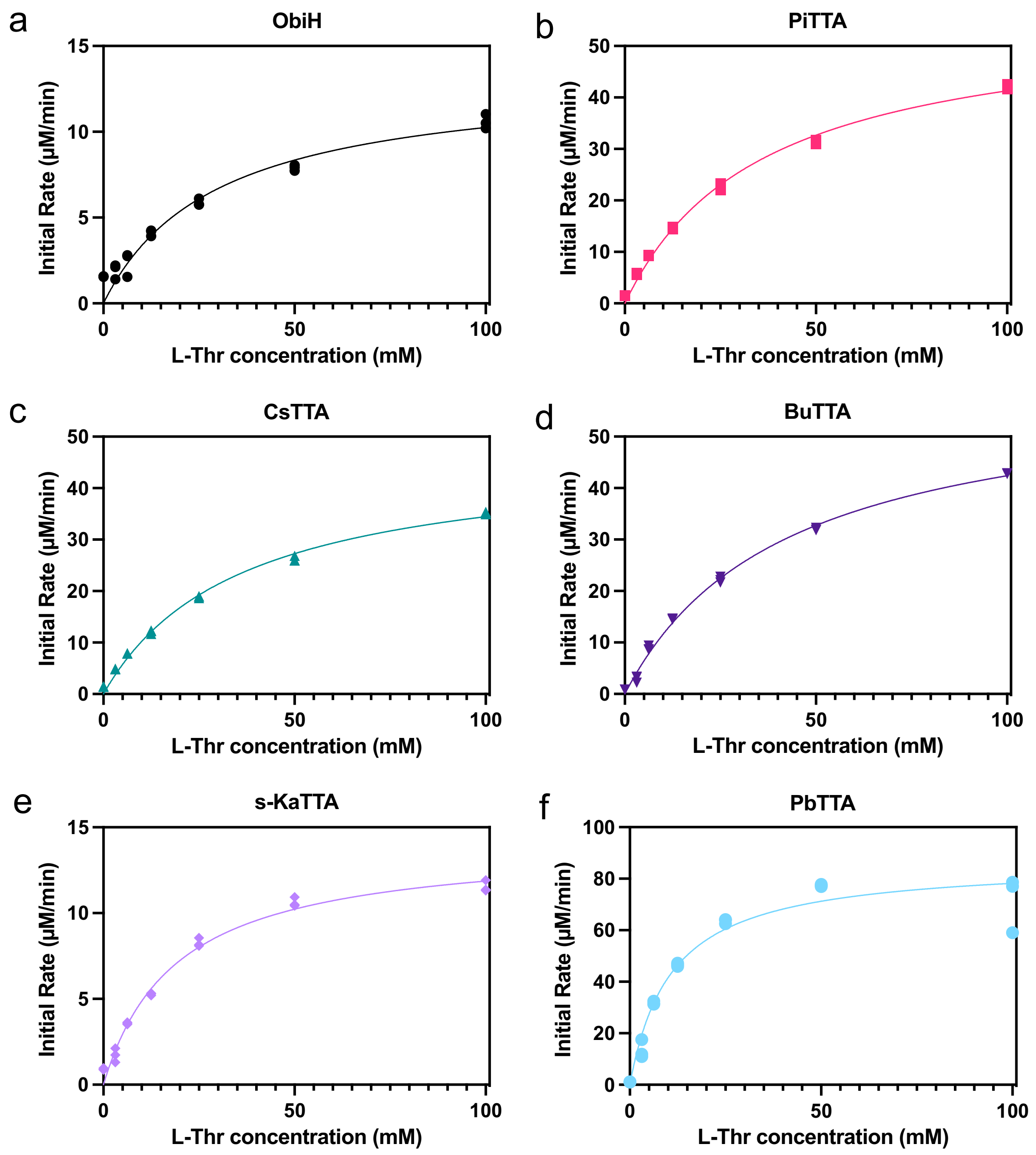

Figure S27: Michaelis-Menten curves for K_M and k_{cat} calculations. Initial rate versus L-Thr concentration to calculate the Michaelis-Menten parameters for each enzyme relative to L-Thr. Each reaction was performed in triplicate with each replicate displayed. The line on each graph is the result of a non-linear regression for Michaelis-Menten analysis and an asymmetric confidence interval calculation using GraphPad Prism to obtain the parameters listed in **Fig. 4b**. The standard TTA-ADH assay was performed using a non-saturating concentration of 1 mM phenylacetaldehyde. Each plot is the measurement for a different TTA with the concentration of initial enzyme listed: (a) ObiH – 0.174 µM (b) PiTTA – 0.203 µM (c) CsTTA – 0.183 µM (d) BuTTA – 0.192 µM (e) s-KaTTA – 0.178 µM (f) PbTTA – 0.200 µM.

Figure S28

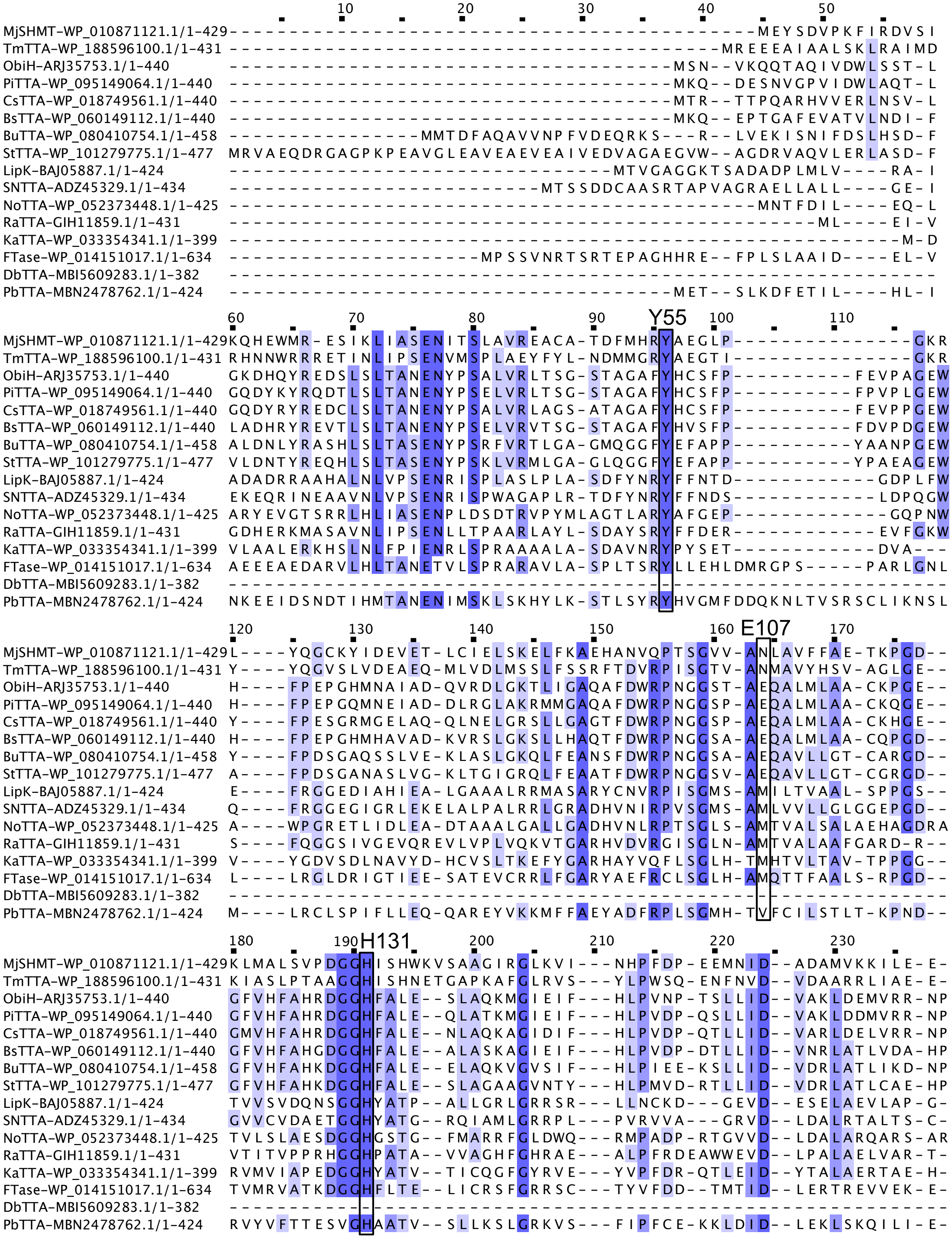
MjSHMT-WP_010871121.1/1-429
TmTTA-WP_188596100.1/1-431
ObiH-ARJ35753.1/1-440
PiTTA-WP_095149064.1/1-440
CsTTA-WP_018749561.1/1-440
BsTTA-WP_060149112.1/1-440
BuTTA-WP_080410754.1/1-458
StTTA-WP_101279775.1/1-477
LipK-BAJ05887.1/1-424
SNTTA-ADZ45329.1/1-434
NoTTA-WP_052373448.1/1-425
RaTTA-GIH11859.1/1-431
KaTTA-WP_033354341.1/1-399
FTase-WP_014151017.1/1-634
DbTTA-MBI5609283.1/1-382
PbTTA-MBN2478762.1/1-424
Y55
E107
H131

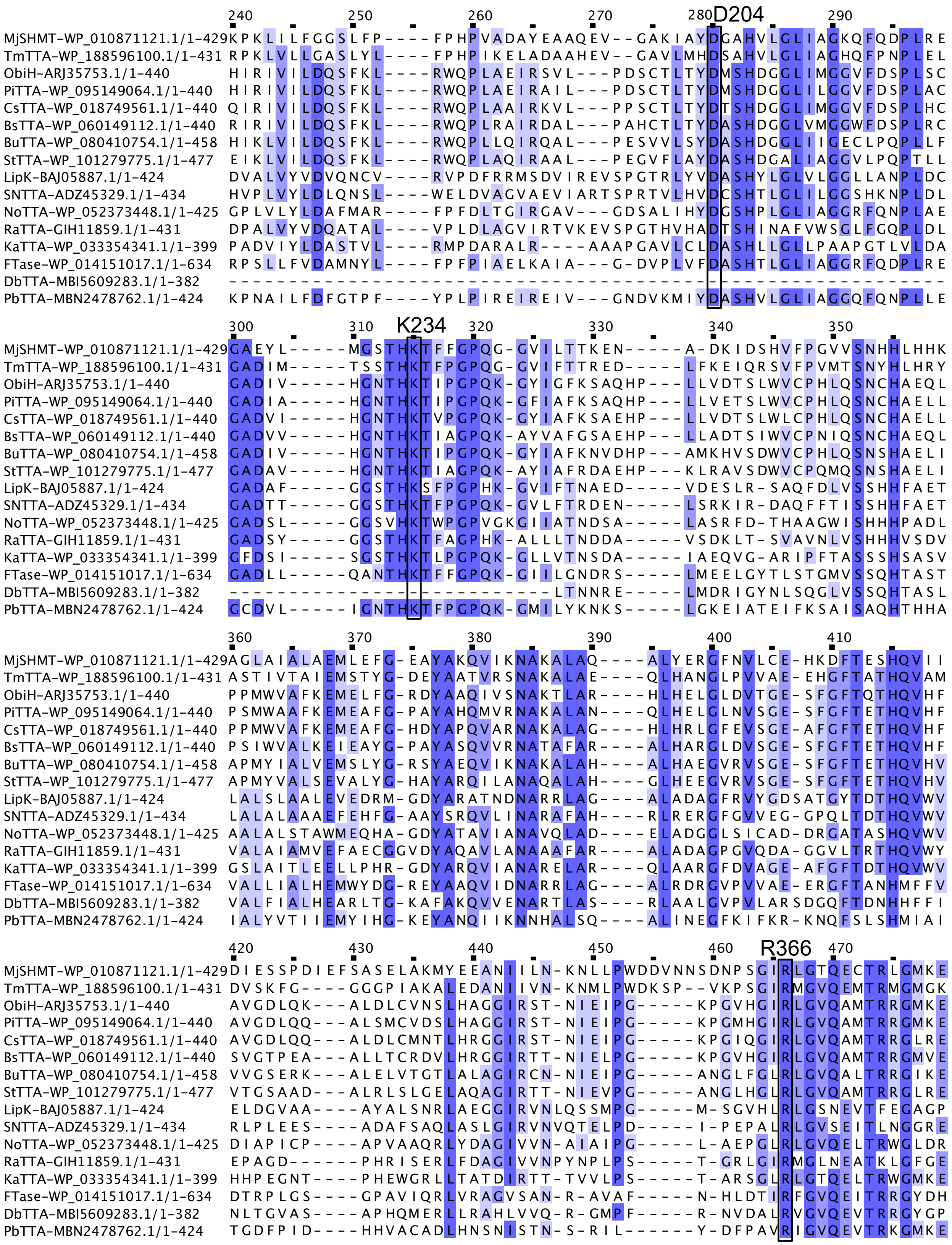
240 250 260 270 280 290
D204
K234
R366
300 310 320 330 340 350
360 370 380 390 400 410
420 430 440 450 460 470
MjSHMT-WP_010871121.1/1-429
TmTTA-WP_188596100.1/1-431
ObiH-ARJ35753.1/1-440
PiTTA-WP_095149064.1/1-440
CsTTA-WP_018749561.1/1-440
BsTTA-WP_060149112.1/1-440
BuTTA-WP_080410754.1/1-458
StTTA-WP_101279775.1/1-477
LipK-BAJ05887.1/1-424
SNTTA-ADZ45329.1/1-434
NoTTA-WP_052373448.1/1-425
RaTTA-GIH11859.1/1-431
KaTTA-WP_033354341.1/1-399
FTase-WP_014151017.1/1-634
DbTTA-MBI5609283.1/1-382
PbTTA-MBN2478762.1/1-424

```
                            480       490       500       510       520       530
MjSHMT-WP_010871121.1/1-429 KEMEEIAEFMKRIAIDKE-KPEKVREDVKEFAKEYSTI--HYSFDEGDGFKYLRFY----
TmTTA-WP_188596100.1/1-431  GEMAAVAELIAKVVIKGV-EPSKVKPEVVELRRGFTKV--RYGFDLSTLGLNCPCLPLL-
ObiH-ARJ35753.1/1-440       KDFEVVARFIADLYFKKT-EPAKVAQQIKEFLQAFPLAPLAYSFDNYLDEELLAAVYQGA
PiTTA-WP_095149064.1/1-440  DDFRRVAGLIADLYFKRT-EPARVASKVKELLGDFPLAPLAYSFDQQIDESRRRLLERGI
CsTTA-WP_018749561.1/1-440  DDFEQVARFIADLHFRKA-DPAGVAAQVAEFLRAFPLAPLHYSFDQELDHELLQSLIGEA
BsTTA-WP_060149112.1/1-440  RDFETVADFIAALCTRKR-TPEDVAPDVETFLGDFPLSPLAFSFDGGMTDALRAALRQGV
BuTTA-WP_080410754.1/1-458  HGMAEVARFLVRLILKNE-SPTAIRNEIASFLESYPINTLHYSLDAHYYTPSGIKLMEEV
StTTA-WP_101279775.1/1-477  PQMREVARLVAKVVLRRA-EPAAVRAEVADLLQHHPLDQLAYSFDSYVDSPAAARLLGEV
LipK-BAJ05887.1/1-424       QAIEELAGALVTARERAL-G----PRTVHEI-RGRFGA--PFYTDPEKLKVEAGL-----
SNTTA-ADZ45329.1/1-434      PAMETLAEIFALVRAGEA-TK--AVDLFQVL-PHEMGE--PYFFTGLPQEAGLFHG----
NoTTA-WP_052373448.1/1-425  DGMTVLTWVLTQLLVHNA-ATAVVAPQMEALRTGLTLPEDRHGLEGFLRACDPQEVSVA-
RaTTA-GIH11859.1/1-431      PEMAELAGLLHGVAVDRI-AVAEAGERVAAM-RQAARP--AYCFSEDVVASKLRELTGA-
KaTTA-WP_033354341.1/1-399  DDMTTVAELLARLLLRGE-QSRSVAADVRDLARSFPGV--AFADRPAPLAVA--------
FTase-WP_014151017.1/1-634  DDLDEAADLVAAVLLERQ-EPERIRPRVAELVGRRRTV--RYTGDPASAAGPPARERYAP
DbTTA-MBI5609283.1/1-382    GEMAQLAEWIASIVIGGA-DPEVVAPAVQAMAKRFDTI--YYTGETVDGKLDL-------
PbTTA-MBN2478762.1/1-424    KDMVQLAKFFKEIILDRK----NISSKIKEFNNKFNSI--EYSLDEIYEKLF--------

                            540       550       560       570       580       590
MjSHMT-WP_010871121.1/1-429 ------------------------------------------------------------
TmTTA-WP_188596100.1/1-431  ------------------------------------------------------------
ObiH-ARJ35753.1/1-440       QR----------------------------------------------------------
PiTTA-WP_095149064.1/1-440  QR----------------------------------------------------------
CsTTA-WP_018749561.1/1-440  LR----------------------------------------------------------
BsTTA-WP_060149112.1/1-440  MR----------------------------------------------------------
BuTTA-WP_080410754.1/1-458  IA----------------------------------------------------------
StTTA-WP_101279775.1/1-477  FR----------------------------------------------------------
LipK-BAJ05887.1/1-424       ------------------------------------------------------------
SNTTA-ADZ45329.1/1-434      ------------------------------------------------------------
NoTTA-WP_052373448.1/1-425  ------------------------------------------------------------
RaTTA-GIH11859.1/1-431      --SGAGVDELAAWLYR--------------------------------------------
KaTTA-WP_033354341.1/1-399  ------------------------------------------------------------
FTase-WP_014151017.1/1-634  PTAPAGHPARPRWIGVRLTPLPEPVTEAECAGAQRLGRLAGAFPHQIDSSGNVSFTSTDG
DbTTA-MBI5609283.1/1-382    -PEIAAPSAKGRWVDYRHLGNDFAMDDTEFSEIRALGAAAGAFPNQTDSTGNVSLRS-GA
PbTTA-MBN2478762.1/1-424    ------------------------------------------------------------

                            600       610       620       630       640       650
MjSHMT-WP_010871121.1/1-429 ------------------------------------------------------------
TmTTA-WP_188596100.1/1-431  ------------------------------------------------------------
ObiH-ARJ35753.1/1-440       ------------------------------------------------------------
PiTTA-WP_095149064.1/1-440  ------------------------------------------------------------
CsTTA-WP_018749561.1/1-440  ------------------------------------------------------------
BsTTA-WP_060149112.1/1-440  ------------------------------------------------------------
BuTTA-WP_080410754.1/1-458  ------------------------------------------------------------
StTTA-WP_101279775.1/1-477  ------------------------------------------------------------
LipK-BAJ05887.1/1-424       ------------------------------------------------------------
SNTTA-ADZ45329.1/1-434      ------------------------------------------------------------
NoTTA-WP_052373448.1/1-425  ------------------------------------------------------------
RaTTA-GIH11859.1/1-431      ------------------------------------------------------------
KaTTA-WP_033354341.1/1-399  ------------------------------------------------------------
FTase-WP_014151017.1/1-634  RLFVTGSGTYIKDLAPGDFVELTGA--EGWTLHCRGDGPPSAEAYLHHLLRERVGARYVV
DbTTA-MBI5609283.1/1-382    RVFVSSSGSYIKHLADGQVVELDAVDPSGELIDYHGAALPSSESLMHFLVYQNVPAGAVV
PbTTA-MBN2478762.1/1-424    ------------------------------------------------------------

                            660       670       680       690       700       710
MjSHMT-WP_010871121.1/1-429 ------------------------------------------------------------
TmTTA-WP_188596100.1/1-431  ------------------------------------------------------------
ObiH-ARJ35753.1/1-440       ------------------------------------------------------------
PiTTA-WP_095149064.1/1-440  ------------------------------------------------------------
CsTTA-WP_018749561.1/1-440  ------------------------------------------------------------
BsTTA-WP_060149112.1/1-440  ------------------------------------------------------------
BuTTA-WP_080410754.1/1-458  ------------------------------------------------------------
StTTA-WP_101279775.1/1-477  ------------------------------------------------------------
LipK-BAJ05887.1/1-424       ------------------------------------------------------------
SNTTA-ADZ45329.1/1-434      ------------------------------------------------------------
NoTTA-WP_052373448.1/1-425  ------------------------------------------------------------
RaTTA-GIH11859.1/1-431      ------------------------------------------------------------
KaTTA-WP_033354341.1/1-399  ------------------------------------------------------------
FTase-WP_014151017.1/1-634  HNHCIPGR-ALETSGALVIPPKEYGSVALAEAVADACQDSQVMYVRRHGLVFWAHSYDEC
DbTTA-MBI5609283.1/1-382    HTHYLLTNQEAADFDVAVIAPQEYASIALARAVAEASKRSRIVYIQKHGLVFWGTDTADC
PbTTA-MBN2478762.1/1-424    ------------------------------------------------------------
```

```
                                 720       730       740
MjSHMT-WP_010871121.1/1-429      -----------------------
TmTTA-WP_188596100.1/1-431       -----------------------
ObiH-ARJ35753.1/1-440            -----------------------
PiTTA-WP_095149064.1/1-440       -----------------------
CsTTA-WP_018749561.1/1-440       -----------------------
BsTTA-WP_060149112.1/1-440       -----------------------
BuTTA-WP_080410754.1/1-458       -----------------------
StTTA-WP_101279775.1/1-477       -----------------------
LipK-BAJ05887.1/1-424            -----------------------
SNTTA-ADZ45329.1/1-434           -----------------------
NoTTA-WP_052373448.1/1-425       -----------------------
RaTTA-GIH11859.1/1-431           -----------------------
KaTTA-WP_033354341.1/1-399       -----------------------
FTase-WP_014151017.1/1-634       LALIEDVRRITG-----------
DbTTA-MBI5609283.1/1-382         LSQVHNFIHNRPNRRAAEAVYAS
PbTTA-MBN2478762.1/1-424         -----------------------
```

Figure S28: Multiple Sequence Alignment. Clustal Omega alignment of representative PLP-dependent enzymes (LipK, FTase, and MjSHMT) and all TTAs in this study colored by % identity. Visualized and aligned using JalView. The residues reported to be important for catalysis and stabilization are highlighted with a black box and the labeled ObiH residue is labeled at the top of the black box.

Figure S29

	ObiH	Y55	E107	H131	D204	K234	R366
Cluster containing	ObiH	93% Y	93% E	93% H	96% D	89% K	93% R
	LipK	100% Y	88% M	100% H	100% D	100% K	97% R
	FTase	94% Y	88% M	88% H	94% D	88% K	100% R
	KaTTA	100% Y	100% M	100% H	100% D	100% K	100% R
	PbTTA	100% Y	40% T	100% H	100% D	100% K	100% R
	SHMT	100% Y	90% N	100% H	100% D	100% K	100% R

Figure S29: Conservation of catalytic and stabilizing residues by cluster. Alignment and identity calculated using JalView via the Clustal Omega alignment web service.

Figure S30

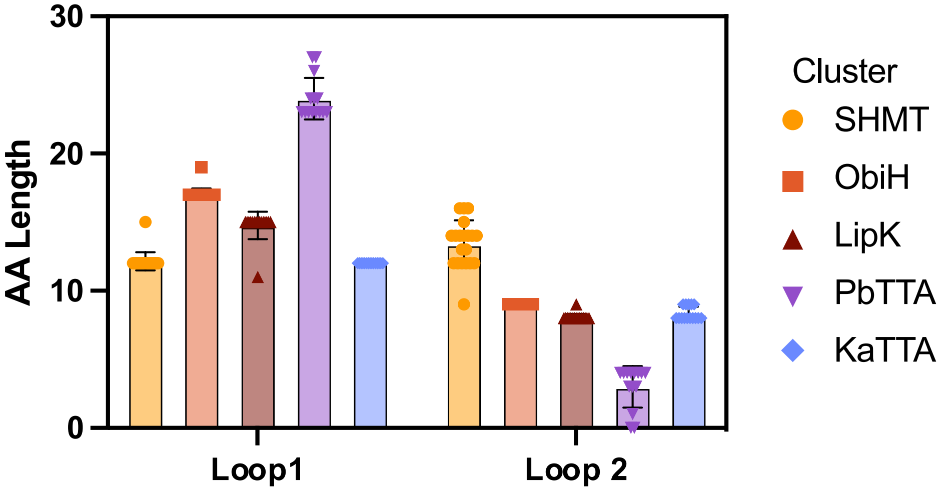

Figure S30: Loop length analysis. The average length for loop 1 (ObiH: Tyr55-Pro71) and loop 2 (ObiH: Glu355-His363) for several clusters. For ObiH, PbTTA, and KaTTA, analysis contains the lengths for every protein in the cluster. For SHMT and LipK, the values are for 25 randomly selected proteins within those clusters. The lengths were defined relative to alignment with ObiH.

Figure S31

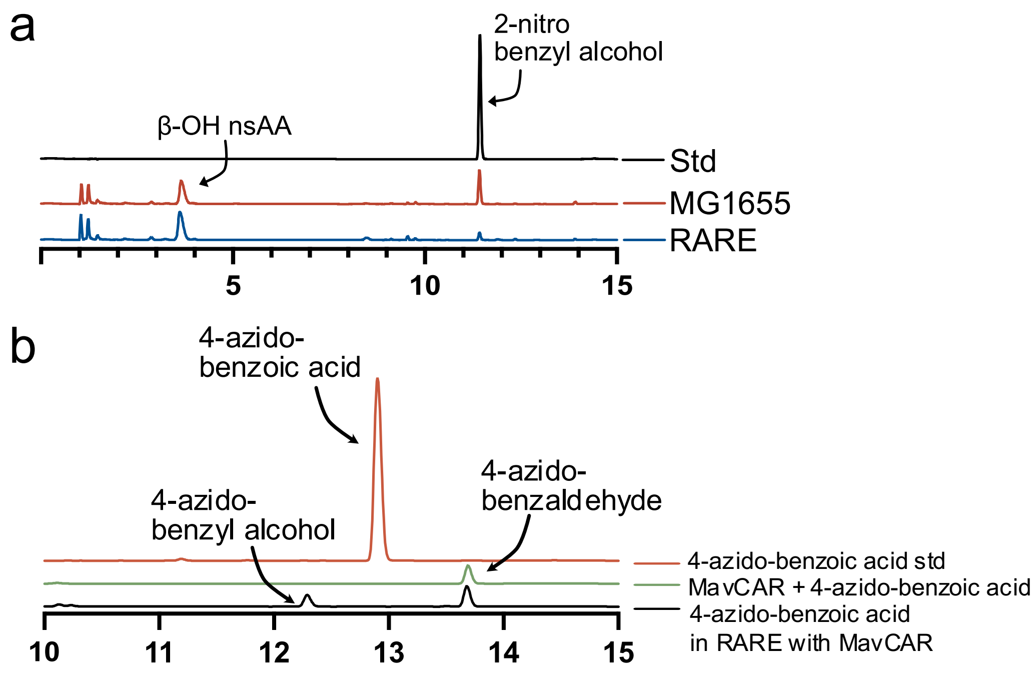

Figure S31: Demonstration of the aldehyde stabilizing effects of the *E. coli* RARE strain for relevant candidate substrates using HPLC. (a) HPLC trace from the production of β-OH-nsAA from **3** with an observed alcohol peak with a retention time at 11 minutes. The samples were collected after 20 h. We observe a smaller alcohol peak in RARE demonstrating some stabilization of the aldehyde group. (b) HPLC trace from the production of 4-azido-benzaldehyde from 4-azido benzoic acid in RARE indicating formation of both an aldehyde peak and a predicted alcohol by-product. MavCAR and 4-azido-benzoic acid is from an in vitro reaction as described in **Fig. 6a** and serves as a standard for the aldehyde since we were unable to purchase an aldehyde standard.

Figure S32

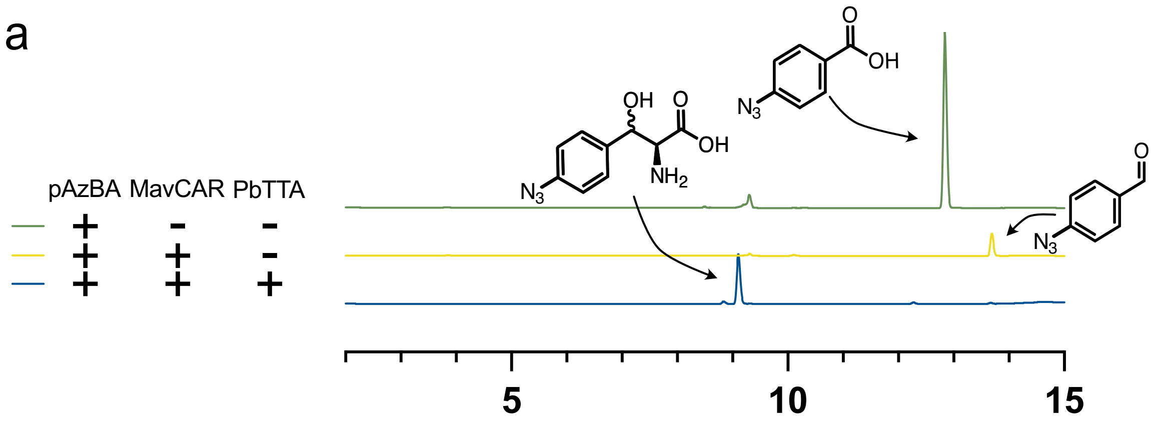

Figure S32: HPLC confirmation for β-OH-nsAA production from 4-azido-benzaldehyde. HPLC traces for acid, aldehyde and β-OH-nsAA product from an in vitro CAR-TTA reaction.

Figure S33

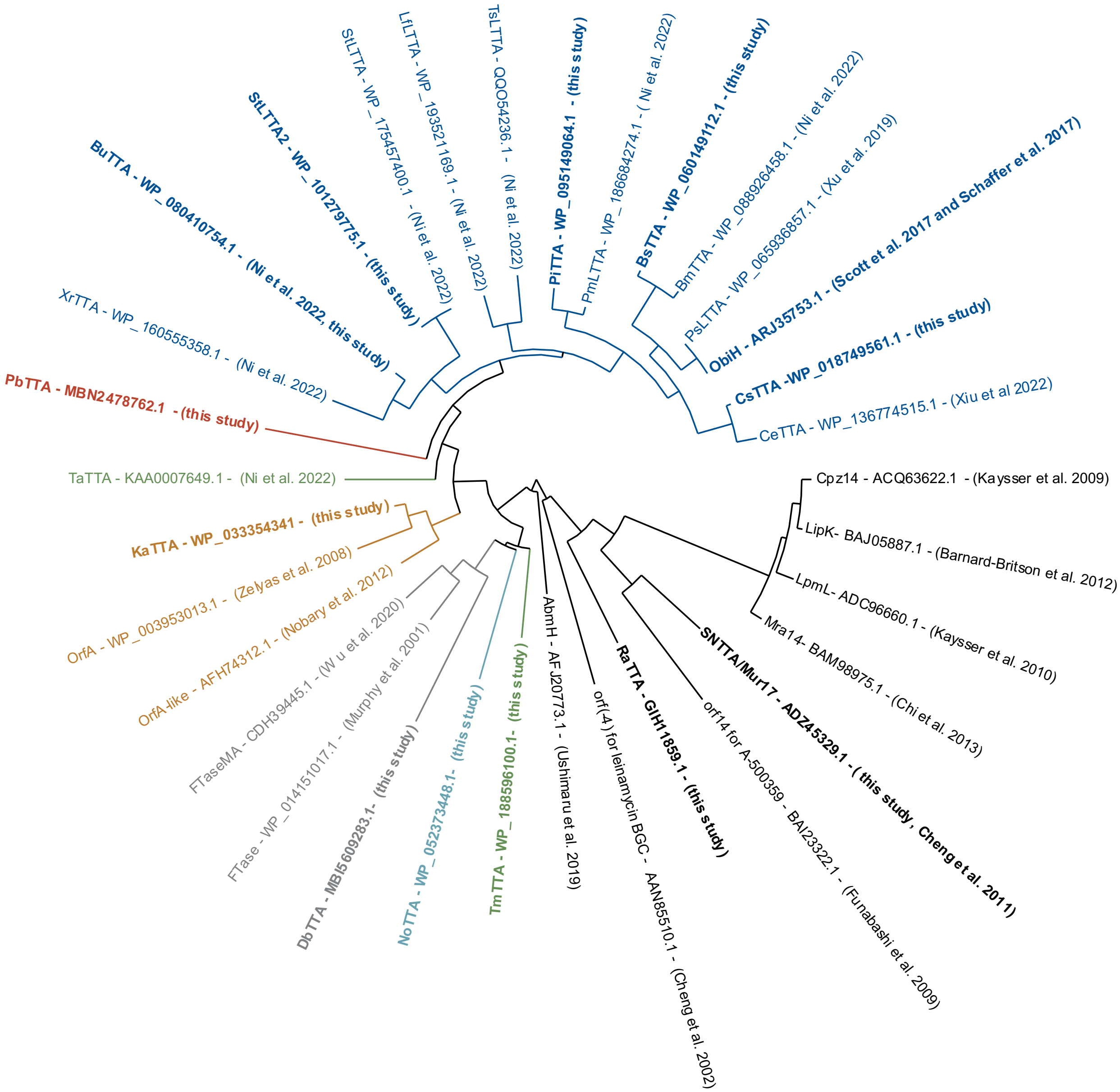

Figure S33: Phylogenetic tree for all published TTAs. Clusters are identified by color: blue is ObiH; red is PbTTA; green is SHMTs; orange is KaTTA; gray is the FTase; teal is NoTTA; black is LipK.[10–23] The phylogenetic tree was generated using the One-click method from phylogeny.fr[24] with the sequences identified in Table S3 and the accession numbers listed in the tree. The bolded TTAs were characterized in this study.

IV. Supplementary References

1. Seiple, I. B., Mercer, J. A. M., Sussman, R. J., Zhang, Z. & Myers, A. G. Stereocontrolled Synthesis of syn-β-Hydroxy-α-Amino Acids by Direct Aldolization of Pseudoephenamine Glycinamide. *Angewandte Chemie International Edition* **53**, 4642–4647 (2014).
2. Kunjapur, A. M., Tarasova, Y. & Prather, K. L. J. Synthesis and Accumulation of Aromatic Aldehydes in an Engineered Strain of Escherichia coli. *Journal of the American Chemical Society* **136**, 11644–11654 (2014).
3. Gopal, M. R. *et al.* Reductive Enzyme Cascades for Valorization of Polyethylene Terephthalate Deconstruction Products. *ACS Catal.* **13**, 4778–4789 (2023).
4. Yang, X. & van der Donk, W. A. Post-translational Introduction of D-Alanine into Ribosomally Synthesized Peptides by the Dehydroalanine Reductase NpnJ. *J Am Chem Soc* **137**, 12426–12429 (2015).
5. Xu, L., Wang, L. C., Su, B. M., Xu, X. Q. & Lin, J. Efficient biosynthesis of (2S, 3R)-4-methylsulfonylphenylserine by artificial self-assembly of enzyme complex combined with an intensified acetaldehyde elimination system. *Bioorganic Chemistry* **110**, (2021).
6. Johannes, T. W., Woodyer, R. D. & Zhao, H. Efficient regeneration of NADPH using an engineered phosphite dehydrogenase. *Biotechnol Bioeng* **96**, 18–26 (2007).
7. Kumar, P. *et al.* L-Threonine Transaldolase Activity Is Enabled by a Persistent Catalytic Intermediate. *ACS Chem. Biol.* **16**, 86–95 (2021).
8. Stothard, P. The sequence manipulation suite: JavaScript programs for analyzing and formatting protein and DNA sequences. *Biotechniques* **28**, 1102, 1104 (2000).
9. Mirdita, M. *et al.* ColabFold: making protein folding accessible to all. *Nat Methods* **19**, 679–682 (2022).
10. Scott, T. A., Heine, D., Qin, Z. & Wilkinson, B. An L-threonine transaldolase is required for L-threo-β-hydroxy-α-amino acid assembly during obafluorin biosynthesis. *Nature Communications* **8**, 1–11 (2017).

11. Xiu, Y., Xu, G. & Ni, Y. Multi-enzyme cascade for sustainable synthesis of l-threo-phenylserine by modulating aldehydes inhibition and kinetic/thermodynamic controls. *Syst Microbiol and Biomanuf* (2022) doi:10.1007/s43393-022-00102-x.

12. Schaffer, J. E., Reck, M. R., Prasad, N. K. & Wencewicz, T. A. β-Lactone formation during product release from a nonribosomal peptide synthetase. *Nature Chemical Biology* **13**, 737–744 (2017).

13. Zelyas, N. J., Cai, H., Kwong, T. & Jensen, S. E. Alanylclavam Biosynthetic Genes Are Clustered Together with One Group of Clavulanic Acid Biosynthetic Genes in Streptomyces clavuligerus. *J Bacteriol* **190**, 7957–7965 (2008).

14. Goomeshi Nobary, S. & Jensen, S. E. A comparison of the clavam biosynthetic gene clusters in Streptomyces antibioticus Tü1718 and Streptomyces clavuligerus. *Can. J. Microbiol.* **58**, 413–425 (2012).

15. Ushimaru, R. & Liu, H. Biosynthetic Origin of the Atypical Stereochemistry in the Thioheptose Core of Albomycin Nucleoside Antibiotics. *J. Am. Chem. Soc.* **141**, 2211–2214 (2019).

16. Cheng, Y.-Q., Tang, G.-L. & Shen, B. Identification and localization of the gene cluster encoding biosynthesis of the antitumor macrolactam leinamycin in Streptomyces atroolivaceus S-140. *J Bacteriol* **184**, 7013–7024 (2002).

17. Funabashi, M. *et al.* Identification of the biosynthetic gene cluster of A-500359s in Streptomyces griseus SANK60196. *J Antibiot (Tokyo)* **62**, 325–332 (2009).

18. Wu, L. *et al.* An unusual metal-bound 4-fluorothreonine transaldolase from Streptomyces sp. MA37 catalyses promiscuous transaldol reactions. *Applied Microbiology and Biotechnology* **104**, 3885–3896 (2020).

19. Murphy, C. D., O'Hagan, D. & Schaffrath, C. Identification of a PLP-Dependent Threonine Transaldolase: A Novel Enzyme Involved in 4-Fluorothreonine Biosynthesis in Streptomyces cattleya. *Angew. Chem. Int. Ed* **40**, (2001).

20. Chi, X. *et al.* The muraminomicin biosynthetic gene cluster and enzymatic formation of the 2-deoxyaminoribosyl appendage. *Medchemcomm* **4**, 239–243 (2013).

21. Kaysser, L. *et al.* Identification and Manipulation of the Caprazamycin Gene Cluster Lead to New Simplified Liponucleoside Antibiotics and Give Insights into the Biosynthetic Pathway *. *Journal of Biological Chemistry* **284**, 14987–14996 (2009).

22. Kaysser, L., Siebenberg, S., Kammerer, B. & Gust, B. Analysis of the liposidomycin gene cluster leads to the identification of new caprazamycin derivatives. *Chembiochem* **11**, 191–196 (2010).

23. Barnard-Britson, S. *et al.* Amalgamation of nucleosides and amino acids in antibiotic biosynthesis: Discovery of an l -threonine: Uridine-5′-aldehyde transaldolase. *Journal of the American Chemical Society* **134**, 18514–18517 (2012).

24. Dereeper, A. *et al.* Phylogeny.fr: robust phylogenetic analysis for the non-specialist. *Nucleic Acids Res* **36**, W465-469 (2008).
