# Supplementary material for: Discovery of l-threonine transaldolases for enhanced biosynthesis of beta-hydroxylated amino acids
Source: Commun Biol. 2023 Sep 11;6:929. doi: 10.1038/s42003-023-05293-0 (PMC10495429; doi:10.1038/s42003-023-05293-0)
Supplement: Supplementary file 3 — Description of Additional Supplementary Files [file 42003_2023_5293_MOESM3_ESM.pdf]

Description of Additional Supplementary Files

File name: Supplementary Data 1

Description: The source data for the figures in the paper.
