# Supplementary material for: Discovery of l-threonine transaldolases for enhanced biosynthesis of beta-hydroxylated amino acids
Source: Commun Biol. 2023 Sep 11;6:929. doi: 10.1038/s42003-023-05293-0 (PMC10495429; doi:10.1038/s42003-023-05293-0)
Supplement: Supplementary file 5 — Reporting Summary [file 42003_2023_5293_MOESM5_ESM.pdf]

nature portfolio

Corresponding author(s): Aditya Kunjapur

Last updated by author(s): Aug 23, 2023

Reporting Summary

Nature Portfolio wishes to improve the reproducibility of the work that we publish. This form provides structure for consistency and transparency in reporting. For further information on Nature Portfolio policies, see our Editorial Policies and the Editorial Policy Checklist.

Statistics

For all statistical analyses, confirm that the following items are present in the figure legend, table legend, main text, or Methods section.

n/a	Confirmed	
☐	☒	The exact sample size (*n*) for each experimental group/condition, given as a discrete number and unit of measurement
☐	☒	A statement on whether measurements were taken from distinct samples or whether the same sample was measured repeatedly
☒	☐	The statistical test(s) used AND whether they are one- or two-sided<br>*Only common tests should be described solely by name; describe more complex techniques in the Methods section.*
☒	☐	A description of all covariates tested
☒	☐	A description of any assumptions or corrections, such as tests of normality and adjustment for multiple comparisons
☐	☒	A full description of the statistical parameters including central tendency (e.g. means) or other basic estimates (e.g. regression coefficient) AND variation (e.g. standard deviation) or associated estimates of uncertainty (e.g. confidence intervals)
☒	☐	For null hypothesis testing, the test statistic (e.g. *F*, *t*, *r*) with confidence intervals, effect sizes, degrees of freedom and *P* value noted<br>*Give P values as exact values whenever suitable.*
☒	☐	For Bayesian analysis, information on the choice of priors and Markov chain Monte Carlo settings
☒	☐	For hierarchical and complex designs, identification of the appropriate level for tests and full reporting of outcomes
☒	☐	Estimates of effect sizes (e.g. Cohen's *d*, Pearson's *r*), indicating how they were calculated

Our web collection on statistics for biologists contains articles on many of the points above.

Software and code

Policy information about availability of computer code

Data collection	Plate-based optical density measurements were collected using Softmax Pro 7.0.3 software with a Spectramax i3x platereader. HPLC data were collected using Agilent ChemStation (Version C.01.10) or OpenLab (Version 2.3) software. Gels and blots were imaged using an Azure c280 imaging system. LC-MS data was collected using a AQUITY Arc UPLC H-class and AQUITY QDa Mass Detector (Waters) with MassLynx (Version 4.2) software. NMR data was collected using Bruker Avance Neo 400 MHz NMR.
Data analysis	HPLC data were analyzed using Agilent ChemStation (Version C.01.10) or OpenLab (Version 2.3) software. Microsoft Excel was used to analyze platereader data, and Graphpad Prism was used to generate plots and calculate standard deviations. Small molecule LC/MS data was analyzed using MassLynx (Version 4.2) software. Additional sequences for threonine transaldolases were identified using a BLASTp search through NCBI and a sequence similarity network was created using the EFI-Enzyme Similarity Tool from the University of Illinois. Sequence similarity networks were visualized using Cytoscope (Version 3.9.1). Protein structures on threonine transaldolases were created using AlphaFold2 via ColabFold and were visualized using PyMOL (Version 2.5.2). NMR data was analyzed with MNova (Version 14.3.3). Sequence alignments were performed and visualized using Jalview (Version 2.11.2.7) and ClustalOWS.

For manuscripts utilizing custom algorithms or software that are central to the research but not yet described in published literature, software must be made available to editors and reviewers. We strongly encourage code deposition in a community repository (e.g. GitHub). See the Nature Portfolio guidelines for submitting code & software for further information.

Data

Policy information about availability of data

All manuscripts must include a data availability statement. This statement should provide the following information, where applicable:

- Accession codes, unique identifiers, or web links for publicly available datasets
- A description of any restrictions on data availability
- For clinical datasets or third party data, please ensure that the statement adheres to our policy

The datasets generated during and/or analyzed during the current study are contained in the published article (and its Supplementary Information) are available from the corresponding author on reasonable request. Source data for figures can be found in Supplementary Data 1.

Research involving human participants, their data, or biological material

Policy information about studies with human participants or human data. See also policy information about sex, gender (identity/presentation), and sexual orientation and race, ethnicity and racism.

Reporting on sex and gender	N/A
Reporting on race, ethnicity, or other socially relevant groupings	N/A
Population characteristics	N/A
Recruitment	N/A
Ethics oversight	N/A

Note that full information on the approval of the study protocol must also be provided in the manuscript.

Field-specific reporting

Please select the one below that is the best fit for your research. If you are not sure, read the appropriate sections before making your selection.

☒ Life sciences ☐ Behavioural & social sciences ☐ Ecological, evolutionary & environmental sciences

For a reference copy of the document with all sections, see nature.com/documents/nr-reporting-summary-flat.pdf

Life sciences study design

All studies must disclose on these points even when the disclosure is negative.

Sample size	Sample sizes were selected based on acceptable convention in the field where 3 replicates is standard practice for plate reader experiments (examples include: (i) doi: 10.1021/ja308185q and (ii) doi: 10.1021/acscatal.2c06219) and HPLC analysis of metabolites produced via fermentation (examples include: (i) doi:10.1038/ncomms11709 and (ii) doi: 10.1038/s41589-020-00684-4). Representative spectra were used for LC-MS analysis based on acceptable convention in the field ((i)doi: 10.1038/s41929-022-00743-0 and (ii)doi: 10.1038/s41586-018-0808-5). No calculation was used to determine sample size, but experiments were repeated to increase replicability.
Data exclusions	No data were excluded.
Replication	All attempts to replicate were successful, and the number of replicates is reported for each experiment. Some full experiments were performed in replicates on separate days, exhibiting similar results with a single day's set of replicates chosen here. Plate reader-based experiments were normally replicated at least twice and most fermentation-based production samples were replicated on 1-2 separate days.
Randomization	Experimental samples using the same strain but coming from different groups were generated from the same source. For this study, randomization was not relevant, as all bacterial cells were analyzed equally.
Blinding	All experiments were unblinded as there would be difficulty fully blinding studies to ensure proper growth and media conditions, but is unlikely researcher bias in would result in replicated effects.

Reporting for specific materials, systems and methods

We require information from authors about some types of materials, experimental systems and methods used in many studies. Here, indicate whether each material, system or method listed is relevant to your study. If you are not sure if a list item applies to your research, read the appropriate section before selecting a response.

Materials & experimental systems

n/a	Involved in the study	
☐	☒	Antibodies
☒	☐	Eukaryotic cell lines
☒	☐	Palaeontology and archaeology
☒	☐	Animals and other organisms
☒	☐	Clinical data
☒	☐	Dual use research of concern
☒	☐	Plants

Methods

n/a	Involved in the study	
☒	☐	ChIP-seq
☒	☐	Flow cytometry
☒	☐	MRI-based neuroimaging

Antibodies

Antibodies used

Horseradish peroxidase-conjugated 6*His, His-tag monoclonal antibody was used from Proteintech (catalog number: HRP-66005; clone number: 21006227)

Validation

Validation was performed by vendor: "Recombinant protein were subjected to SDS PAGE followed by western blot with HRP-66005 (6*His, His-Tag antibody) at dilution of 1:10000 incubated at room temperature for 1.5 hours."
